# Self-acupressure for patients with breast cancer experiencing aromatase inhibitor-associated musculoskeletal symptoms: Protocol for the AcuAIM randomized pilot trial

**DOI:** 10.1371/journal.pone.0311044

**Published:** 2025-01-22

**Authors:** N. Lynn Henry, Kelley M. Kidwell, Stephanie Kozar, Sara Snyder, Suzanna M. Zick

**Affiliations:** 1 University of Michigan Medical School, Ann Arbor, MI, United States of America; 2 University of Michigan School of Public Health, Ann Arbor, MI, United States of America; University Hospital Cologne: Uniklinik Koln, GERMANY

## Abstract

**Background:**

Aromatase inhibitors (AI) reduce hormone receptor-positive breast cancer recurrence risk by about 50%. However, half of AI-treated postmenopausal women report new or worsened musculoskeletal symptoms (AIMSS), and 20% discontinue therapy prematurely. Acupuncture is effective for reducing symptoms, but many women are not able to access acupuncture therapy. We hypothesize that self-administered acupressure will reduce AIMSS.

**Materials and methods:**

Postmenopausal women who have been receiving treatment with an AI for more than 3 weeks but less than 2 years, and who report new or worsened joint pain or myalgias since starting AI therapy with worst pain of at least 4 out of 10 on a numerical rating scale, are eligible. Fifty participants will be enrolled and randomized 1:1 to treatment with true or sham acupressure for 12 weeks. Participants will self-apply pressure for 3 minutes to each of the 9 acupoints daily. All participants will complete a pain assessment weekly, and a battery of symptom questionnaires every 6 weeks. Optional stool samples will be collected after 0 and 12 weeks of acupressure to examine changes in the gut microbiome. The primary endpoint is change in worst pain on the Brief Pain Inventory-Short Form with 12 weeks of the acupressure intervention, evaluated with generalized estimating equations.

**Conclusion:**

Determination that self-administered acupressure reduces AIMSS in this randomized phase 2 pilot trial will lead to a larger randomized phase 3 clinical trial to confirm the efficacy of self-acupressure. Reduction of AI-related arthralgias may improve persistence with breast cancer therapy, breast cancer outcomes, and quality of life for AI-treated patients.

**Trial registration:**

Clinicaltrials.gov NCT06228768.

## Introduction

### Aromatase inhibitors

Breast cancer is the most common cancer diagnosis among women in the United States, affecting one in eight women in her lifetime. About 80% of breast cancers diagnosed in postmenopausal women and almost all breast cancers diagnosed in men are hormone receptor positive (HR+). For these patients, daily oral adjuvant endocrine therapy for five to ten years following primary treatment (surgery, radiation, chemotherapy) is indicated [1]. Third generation aromatase inhibitors (AI) are the preferred endocrine treatment for postmenopausal women with early-stage HR+ breast cancer. Aromatase is the key enzyme that converts androgens to estrogens. In postmenopausal women, aromatase is only expressed in non-glandular tissues including fat, liver, brain, and breast tissues [2]. AIs prevent the biosynthesis of estrogen in these tissues, thus preventing the proliferation of HR+ breast cancer cells. Large-scale comparative effectiveness trials demonstrated the superiority of AIs over other endocrine treatment (i.e., tamoxifen), in terms of prolonged disease-free survival, rates of distant metastasis, and contralateral breast cancer, as well as a preferable toxicity profile [3].

### AI toxicity

AI-associated musculoskeletal symptoms (AIMSS) affect about half of the 200,000 patients who start AI therapy each year in the United States [4]. Musculoskeletal symptoms, including arthralgias, myalgias, and joint stiffness, are the primary symptoms reported by AI-treated patients. Despite considerable research, the etiology of AIMSS remains poorly understood [4–8]. Postulated mechanisms include inflammation and reductions in naturally anti-nociceptive properties of estrogen [9, 10]. While multiple randomized clinical trials of AIMSS treatments have been conducted, only a few interventions including acupuncture and duloxetine have demonstrated improvement in musculoskeletal pain and stiffness, and these interventions are not effective for all patients [11, 12].

Despite well-established clinical benefits of long-term AI treatment, more than 20% of patients discontinue AI therapy prematurely, primarily because of toxicity of therapy. Early treatment discontinuation can increase risk of breast cancer recurrence by 45–50% [4, 13–15]. While the primary toxicity of therapy is AIMSS, AI-treated patients also report bothersome vasomotor and gynecologic symptoms, as well as insomnia, fatigue, anxiety, and depression. In addition, studies have shown an increase in inflammatory markers in patients with co-existing symptoms, supporting an underlying inflammatory mechanism [10]. Therefore, well-tolerated and effective interventions to improve AI-associated symptoms, primarily AIMSS, are urgently needed.

### Acupuncture and AIMSS

Acupuncture is a traditional Chinese medicine (TCM) that involves insertion of fine, single-use, sterile needles into acupoints throughout the body. It is a nonpharmacologic modality used for treating a variety of conditions, including pain. A multicenter randomized, controlled trial of true acupuncture, sham acupuncture, and wait-list control for treatment of AIMSS was conducted by the SWOG Cancer Research Network [12]. In the trial, participants randomized to true acupuncture received treatment at standardized acupoints, and could also receive treatment to up to 3 additional acupoints depending on sites of pain [16]. From baseline to 6 weeks, the mean worst pain score decreased by 2.05 points in the true acupuncture group, by 1.07 points in the sham acupuncture group, and by 0.99 points in the wait-list control group. Between the true and sham acupuncture groups, the adjusted difference was 0.92 points (95% CI 0.20–1.65, p = .01). Based on these results, acupuncture is an effective therapy for treating AIMSS. However, uptake is incomplete because of the cost of acupuncture, limited availability of acupuncturists, and for some patients, discomfort with needles [17].

### Acupressure

Acupressure is a technique derived from acupuncture and is also used to address health issues. It is a component of TCM in which pressure is applied to specific acupoints on the body using a finger, thumb, or small device, rather than stimulating the acupoints using needles. Acupressure has shown promise for treating fatigue and other symptoms in cancer patients and survivors [16–20]. It can be delivered by a practitioner or can be self-administered.

Zick and colleagues performed a randomized clinical trial of relaxing and stimulating self-acupressure in fatigued breast cancer survivors [18]. In this study, 288 participants were randomized to one of three groups: relaxing acupressure (n = 94), stimulating acupressure (n = 90), or usual care (n = 86). Participants performed acupressure daily for 6 weeks in both acupressure groups. At 6 weeks, 66% of participants receiving relaxing acupressure, 61% receiving stimulating acupressure, and 31% receiving usual care reported normal levels of fatigue as assessed with the Brief Fatigue Inventory [19, 20]. Improvements in fatigue persisted over the next 4 weeks after the completion of acupressure treatment in both arms. This trial also found that in the 89 women with chronic pain at baseline (>3 on a 10-point pain visual analog scale) there was a significant decrease in pain severity and interference at both the 6 and 10 week time points in the relaxation acupressure group compared to the usual care group (relaxing acupressure at 6 weeks had a 1.3 ± 2.0 point decrease in pain, whereas those receiving usual care had a 0.1 ± 2.0 point increase in pain (p = 0.003)) [21–23]. Mean adherence to performing acupressure treatments, defined as completing at least 34 of 42 total treatments as recorded in logbooks, was 79.8% (standard deviation 22.9%), and 53 out of 74 women (72%) completed at least 70% of their acupressure treatments [18].

### Mechanism underlying AIMSS development

Despite considerable research the etiology of AIMSS remains uncertain [4–6]. Postulated mechanisms include inflammation and reductions in naturally anti-nociceptive properties of estrogen, although the actual mechanism remains unclear. Researchers have investigated associations between both clinical factors and genetic and biochemical biomarkers and development of musculoskeletal symptoms [4, 24]. In particular, we have demonstrated that pre-existing nociplastic pain and greater increase in pain sensitivity with AI therapy are associated with shorter time to AI discontinuation because of symptoms [5, 25]. However, we have been unable to identify associations between systemic inflammatory cytokine concentrations and development of AIMSS [6]. To date, it has been challenging to elucidate the etiology of AIMSS or to identify effective management options.

The gut microbiota plays a critical role in human health, including metabolism, obesity, and joint injury [26]. Disruption of the gut microbiota is influenced by many factors, including diet and medications, and has been closely linked to the development of chronic inflammation [27]. Studies have shown that the gut microbiome is influenced by estrogen, and also influences estrogen levels [28]. To date, there have been few studies of the impact of estrogen deprivation on the gut microbiome, or its subsequent effects. In a mouse model of polycystic ovarian syndrome, AI-treated mice had a reduction in fecal microbial richness and alpha diversity over time, including increases in bacterial species associated with inflammation [29]. However, it is unknown either how estrogen deprivation influences the microbiome or whether there are associations between changes in the microbiome and development of treatment-emergent toxicity. Also, acupressure and acupuncture have been associated with an increased abundance of anti-inflammatory bacteria in the gut [30, 31]. Therefore, during the conduct of this study we will request that participants submit optional stool samples so that we can determine whether there are changes in the gut microbiome that occur during treatment with acupressure, and explore whether the changes are associated with reduction in AIMSS symptoms.

## Materials and methods

### Trial design

**Fig 1:**
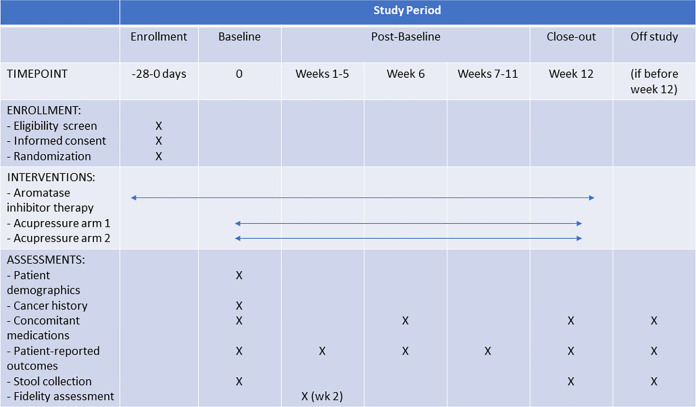
Overview of trial design. Standard Protocol Items: Recommendations for Interventional Trials (SPIRIT) schedule of enrollment, interventions, and assessments.

The AcuAIM clinical trial is a randomized, controlled, pilot trial designed to test whether 12 weeks of daily true acupressure is superior to 12 weeks of daily sham acupressure in reducing pain in patients with AIMSS (Fig 1, S1 and S2 Files). Following registration and baseline assessment, participants will be randomly assigned 1:1 to the intervention arms using random block randomization. Daily, participants will complete an electronic study diary to report use of the study intervention and AI therapy. Participants will have phone assessments to record current prescription and over-the-counter supplement use every 6 weeks for 12 weeks, including ongoing use of AI therapy. The protocol has been approved by the University of Michigan Institutional Review Board (HUM00241228) and enrollment started in March 2024 (clinicaltrials.gov NCT06228768). Enrollment is anticipated to take about 12 months.

### Randomization procedures

Participants, clinicians, and study personnel will be blinded to group allocation, with the exception of a single research assistant who is assessing fidelity with the intervention via video call at week 2 and is aware of treatment assignment. The unblinded research assistant has no other contact with study participants, and will not be involved in the data analysis. The study member who is responsible for uploading the application onto the computer tablets will label the tablets different colors depending on whether the application with the true or sham acupressure is included. The research assistant who interacts directly with the study participants is not aware of which color corresponds with which version of the application. The randomization list generated prior to the start of the study includes study identification numbers and corresponding colors, and the research assistant uses this list when sending a computer tablet to each participant.

### Participants

Eligibility criteria for participants include: (1) female or male; (2) age ≥18 years; (3) taking the currently prescribed AI therapy (anastrozole, exemestane, or letrozole) for adjuvant or palliative treatment of breast cancer or for chemoprevention for at least 3 weeks and no more than 2 years at the time of enrollment; (4) planning to take the same AI medication for at least 12 weeks; (5) new or worsening joint pain and/or myalgias since starting the AI therapy, with worst pain score of at least 4 out of 10 on the Brief Pain Inventory (BPI) over the 7 days prior to enrollment; [32] (6) completion of radiation therapy, if given, for treatment of breast cancer; (7) completion of chemotherapy, if given; (8) patients receiving treatment with NSAIDs, acetaminophen, opioids, duloxetine, cannabinoids, gabapentin, and/or pregabalin must have been taking a stable dose for at least 30 days prior to enrollment if they plan to continue the drug during study participation, or discontinue the medication at least 7 days before initiation of study treatment; (9) able to read, understand, and self-complete questionnaires in English; (10) able to access WiFi/internet and willing to use an email account or download and use the MyDataHelps app; (11) no use of acupuncture or acupressure in the past year, or planned use of acupuncture during study participation; (12) no use of systemic or transdermal estrogen during study participation; (13) no planned surgery during the 12-week study period; (14) no concurrent medical or arthritis disease that could confound or interfere with evaluation of pain or efficacy; and (15) no prior or concurrent malignancy whose natural history or treatment has the potential to interfere with the safety or efficacy assessment of the investigational regimen. Concurrent treatment with gonadotropin-releasing hormone agonist (GnRHa) therapy, anti-osteoclast therapy, anti-HER2 therapy, poly-ADP ribose polymerase (PARP) inhibitor therapy, and cyclin-dependent kinases 4 and 6 (CDK4/6) inhibitor therapy is permitted.

### Recruitment procedures

Participants will be recruited from breast cancer clinics at the University of Michigan Rogel Cancer Center, as well as through online platforms such as UMHealthResearch.org and clinicaltrials.gov and through communication with patient advocacy groups and other cancer-focused community organizations. Since the study procedures are entirely virtual, participants can be anywhere in the United States during study participation. Study personnel will approach potentially eligible patients and also discuss the trial with those who reach out to the study team with interest in participation. Those who are interested in participating will provide written informed consent electronically prior to undergoing any protocol-directed procedures. In addition, study participants will provide additional written consent electronically for the optional collection and use of biological samples.

### Intervention

#### Self-acupressure

**Fig 2:**
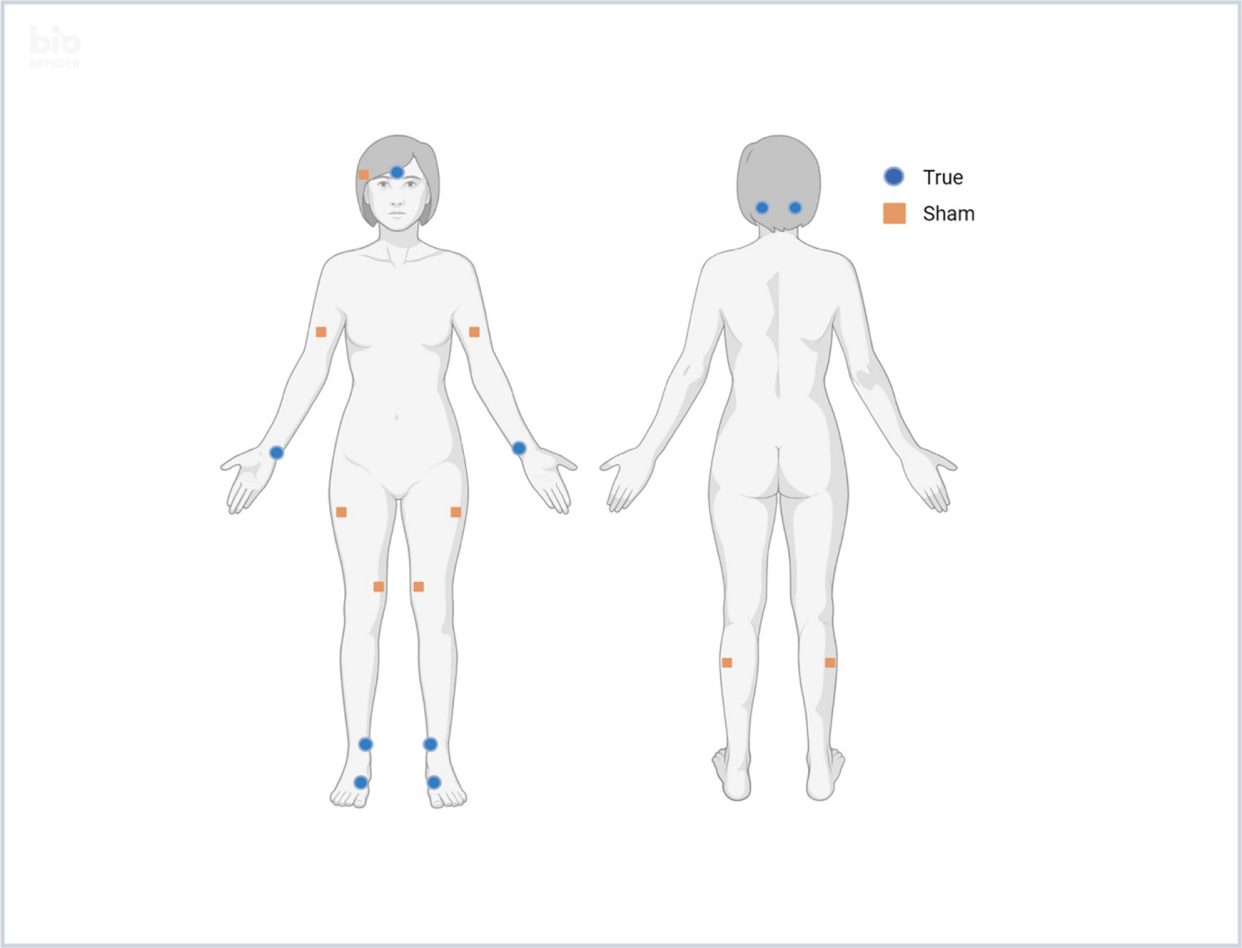
Points where pressure will be applied daily, by study arm. Blue circles denote the acupoints to be stimulated by participants randomized to the true acupressure arm. Orange squares denote the locations to be stimulated by participants randomized to the sham acupressure arm. Created with biorender.com.

As noted above, acupressure is a technique derived from acupuncture in which pressure is applied to specific acupoints on the body using a finger, thumb or small device, rather than stimulating the acupoints using needles. This trial will compare two sets of acupoints, each of which has 4 acupoints performed on both the right and left sides of the body and one unilateral acupoint, for a total of 9 acupoints (Fig 2). One set (“true”) of relaxing acupressure acupoints has previously been shown to be effective for treating fatigue and pain in patients with breast cancer, and includes Yin tang (unilateral), Anmian (bilateral), Heart 7 (bilateral), Spleen 6 (bilateral), and Liver 3 (bilateral) [18, 22]. The second set (“sham”) has points that are not located on meridians and are at least one inch away from meridians and the acupoints in the first set. This set includes a unilateral pressure point above the right ear, bilateral points on the muscle of the upper arm, bilateral points on the upper lateral thigh, bilateral points on the lower medial thigh, and bilateral points on the lateral gastrocnemius. Each acupoint will be stimulated for 3 minutes continuously every day, for a total of 27 minutes of acupressure therapy daily for 12 weeks. Participants will complete an electronic diary daily to record acupressure therapy, including the length of treatment and whether any breaks were included.

Participants will be educated about how to perform acupressure using a study-specific mobile application (app) developed at our institution and provided to participants on a tablet [33]. For administration of the acupressure, participants will be instructed to make small clockwise or counterclockwise circles with the AcuWand shell or with their index finger, thumb, or a pencil eraser, with sufficient pressure to evoke a “de qi” sensation, which is described as a dull ache, tingling, and soreness [34]. The AcuWand shell, (Arbor Medical Innovations, Ypsilanti, Michigan) which was designed to be comfortable to hold and to minimize strain in the hands or fingers for applying the acupressure [33], will be provided to participants. It incorporates an ergonomic handle so the user can easily grasp the device; the user can apply pressure through the sprung probe which has a soft rubber tip.

Fidelity with the intervention will be assessed by research staff during week 2 (±1 week) using a video conferencing call. The research staff conducting this assessment will not be blinded to study arm. Participants will be asked to identify the location, stimulation technique, and amount of pressure they are applying to the acupoints. The number of acupoints correctly located will be recorded, as well as the adequacy of stimulating the acupoints, recorded as yes or no. In particular, use of the correct circular motion, maintenance of contact with the skin, and appropriate application of pressure such that the nail blanches will be assessed, and the percentage of correct responses will be calculated.

### Concomitant care during trial participation

Participants who are taking analgesics at study entry should continue to take the same dose of medication throughout the 12 week study period; medication use will be assessed every 6 weeks. During study participation, participants must avoid acupuncture therapy, as well as use of systemic or transdermal estrogen products.

### Discontinuing protocol participation

Participants will stop protocol-directed treatment if they: (1) have new cancer, cancer recurrence, or cancer progression; (2) discontinue the currently prescribed AI therapy for at least 7 days; (3) experience unacceptable toxicity; (4) have a delay of 14 consecutive days of study treatment for any reason; (5) initiate acupuncture therapy; or (6) wish to discontinue study participation.

### Patient-reported outcomes

Each week during study participation, participants will complete a brief 2 item electronic questionnaire about worst and average pain. At baseline and every 6 weeks, participants will complete questionnaires electronically about symptoms (Table 1). Prior to study participation, participants will also complete an assessment of expectancy of benefit from acupressure, and after 6 weeks participants will complete an assessment of acupressure sensation. After completion of the study, participants will complete a questionnaire about which study arm they think they were randomized to, whether the treatment was worth it, and whether they would recommend the treatment to others.

**Table 1:** Patient-reported questionnaires.

Instrument	Description	Timepoints
Brief Pain Inventory-Short Form (BPI)	14-item pain assessment tool to assess joint pain, joint stiffness, and symptom burden over the past 7 days. There are subscales for worst pain, average pain, and pain interference rated on scales of 0–10 with 10 signifying the worst symptoms. This scale has been validated for use in patients with cancer pain, and has been used in multiple studies of patients with AIMSS. Only the worst and average pain items will be assessed weekly [11, 12, 32].	*Baseline and weeks 6 and 12 (±2 days); 2-item weekly assessment (±2 days)*
PROMIS-29+2 Profile v.2	31-item questionnaire to assess patient-reported symptoms in 9 PROMIS domains (pain, pain interference, fatigue, sleep disturbance, physical functioning, depression, anxiety, ability to participate in social roles and activities, and cognitive function-abilities) over the past 7 days. Calculated raw scores for each domain are converted to a T-score, with mean 50 and standard deviation 10. Higher T scores represent more of the concept being measured [35].	*Baseline and weeks 6 and 12 (±2 days)*
Global Ratings of Change (GRC)	Single item measure to assess the overall change in pain and stiffness since starting study treatment. It is a 7-point Likert scale from -3 to +3.	*Baseline and weeks 6 and 12 (±2 days)*
Demographics	5-item survey to collect information about sex, race/ethnicity, education, marital status, and income	*Baseline*
Mao Expectancy of Treatment Effect (METE)	4-item questionnaire rated on a scale of 1–5 (from total disagreement to total agreement), which assesses a patient’s expectation that acupressure will relieve her AIMSS symptoms [36]. It had good internal consistency (Cronbach’s α = 0.95) in a prior study of acupuncture in patients with cancer [37].	*Baseline*
Fibromyalgia Survey (FM)	A combined measure of widespread pain (body map of painful sites) and symptom severity (e.g., fatigue, cognitive problems, headache, poor mood, scores range 0–12), as a self-reported proxy of centralized pain. This is a continuous measure with scores ranging from 0–31 [38, 39].	*Baseline*
Massachusetts General Hospital Acupuncture Sensation Scale (MASS)	A 13-item measure that consists of twelve predefined descriptors and one subjective component specified by the subjects in their own words to measure levels of *de qi* sensations [34].	*Week 6 (±2 days)*
Post-intervention survey	5-item measure to assess what type of acupressure participants thought they were performing (true acupressure or sham acupressure), whether they felt the benefits they received were worth the time spent performing acupressure, whether they would recommend this intervention to others, and whether the AcuWand was helpful for administering acupressure.	*Week 12 (±2 days) or end of treatment*

Questionnaires will be completed electronically by enrolled participants.

### Correlative studies

Participants will be offered participation in optional stool sample collection. Participants will collect stool samples at home before starting acupressure and after 12 weeks using a provided kit and will mail samples to the clinic. Samples will be analyzed using a standard 16S protocol to target and amplify the V4 region [40], so that changes in bacterial phyla and genera and Shannon diversity indices with acupressure therapy can be explored. Acupressure and acupuncture have previously been shown to impact the gut microbiome [30].

### Statistical analyses

#### Sample size

A total of 50 patients will be enrolled. For the sample size calculation, we assumed a standard deviation of difference in worst pain score between treatment groups of 2.3 points based on data from a prior acupuncture clinical trial [41]. With a sample size of 44 evaluable participants (22 evaluable per arm), we will therefore have 80% power to detect a reduction of 2 points in worst pain on the BPI at 12 weeks, assuming a two-sided 5% significance level and a simplified two-sample t-test. A 2-point improvement in pain is considered a minimal clinically important difference [42]. To account for potential dropout, we are planning to accrue 50 participants to ensure that 44 participants are evaluable for the primary endpoint.

#### Primary outcome

The primary endpoint is change in worst joint pain (range 0–10) with 12 weeks of the intervention. An intent to treat analysis will be used. A linear model with the outcome being weekly worst joint pain scores from week 1 to 12 using generalized estimating equations with robust variance with covariates of baseline worst joint pain score, treatment group, time, and a time by group interaction will determine the difference between true acupressure and sham acupressure. We will use an identity link function employing a transformation if needed on the outcome. We will assess the best functional form of time (e.g. linear, quadratic, cubic, splines) in our model. The primary analysis of interest is a planned contrast of the 12 week by treatment group interaction to understand if average worst pain, adjusted for baseline worst pain, differs between the two treatment groups. We will also assess the rate of change in each group over time.

#### Secondary outcomes

The proportion of patients who have at least a 2-point reduction in worst and average pain from baseline to 12 weeks will be reported with the corresponding exact binomial confidence intervals in each treatment group. The proportions will be compared using a chi-square or Fisher’s exact tests for each outcome. Linear models using generalized estimating equations will assess the change in average and worst pain and pain interference over time between groups similar to the primary endpoint.

Descriptive statistics including mean, standard deviation, median, interquartile range, t tests, and chi squared tests will be used to describe all other patient-reported outcomes (sleep disturbance, fatigue, physical function, anxiety, depression, and cognitive function over time), as appropriate. Patient reported outcomes will be analyzed using linear models with generalized estimating equations with robust variance similar to the primary outcome. Normalizing transformations will be used if necessary to ensure accurate model fit.

We will assess the success of blinding by comparing the proportion of participants who believed they had received true acupressure across groups using a chi-square test. Adherence to the intervention will be assessed using self-report throughout the trial and reported using descriptive statistics. Adherence will be calculated based on number of days acupressure is performed and number of minutes of acupressure performed each day. The proportion of participants who are fully adherent will be compared between groups using a chi-square test. Safety will be assessed throughout the trial and adverse events will be reported using descriptive statistics.

#### Exploratory outcomes

The first exploratory outcome is examination of the association between expectation of response to acupressure based on the METE questionnaire and observed response to acupressure based on change in BPI worst pain score. This association will be analyzed using Spearman correlation.

In the second exploratory outcome, the gut microbiota at baseline and after 12 weeks will be analyzed by group. Change in bacterial phyla and genera proportions and Shannon diversity indices over time will be examined using Wilcoxon rank sum tests to compare groups. Principal components analysis will be conducted to display the microbiome space between compare groups. Differences in structure among groups will be assessed using permutational multivariate analysis of variance (PERMANOVA).

There is no adjustment for multiple comparisons for all secondary and exploratory endpoints as these are hypothesis generating.

#### Missing data

We plan to reduce missing data through monitoring of submission of electronic patient-reported outcomes data and reaching out to participants via text, email, or phone if questionnaires are not completed within protocol-defined assessment windows. We will record all reasons for study withdrawal and missing data. We will assess missing data across study groups, covariates and time points and use multiple imputation via chained equations if it is appreciable.

### Data availability

After completion of the study and publication of the results, de-identified data will be available to other investigators upon reasonable request.

## Discussion

Tremendous advances have been made in treatments for cancer, including with anti-endocrine therapy for hormone receptor positive breast cancer. However, a substantial proportion of patients are unable to tolerate the treatment and discontinue treatment prematurely. Therefore, identifying effective and accessible therapies for symptom management is critically important to improve disease outcomes and maintain quality of life for patients. Acupuncture has been shown to reduce AIMSS, but uptake has been limited because of cost and availability. Acupressure may be a cost-effective alternative, since it can be self-administered at home by patients and does not require any specialized equipment, although it requires a not insignificant amount of time daily to administer the therapy. We are therefore conducting this pilot clinical trial to determine if self-administered acupressure may be an effective and acceptable alternative for managing these aggravating symptoms.

### Acupressure intervention

For this clinical trial we are employing an app and a device that has previously been used in other clinical trials of self-acupressure [33]. The mobile app is an easy to use platform to enable trial participants to perform acupressure at home with high levels of fidelity and adherence. If successful, use of this app will permit testing in a larger, multicenter trial without needing to employ a large cadre of study coordinators for teaching self-acupressure. In addition, use of the app will allow for easier dissemination of the intervention in the future.

We are also providing participants with the shell of a device, called the AcuWand, which is ergonomically easier to hold for application of pressure, although because of technical difficulties the version of the device being used in this trial lacks the ability to quantify and capture applied pressure. For this population of postmenopausal women and older men, many of whom have pre-existing osteoarthritis and who additionally have joint pain and stiffness as a result of AI therapy, holding the AcuWand to apply pressure will likely be easier than holding a smaller device, such as a pencil, or applying pressure directly with a finger or thumb. To assess fidelity, participants will have a video call with study personnel about 2 weeks after starting study participation to confirm that they are performing acupressure correctly. We will also query participants at the end of the study to determine whether they felt the AcuWand was useful.

### Acupoint selection

One key aspect of the study design was deciding which acupoints to test in the experimental and the sham cohorts. The published acupuncture trials for treatment of AIMSS used a standardized set of full body, auricular, and up to 4 sets of joint-specific acupuncture points, administered twice a week for 6 weeks then weekly for 6 weeks [16]. The number of acupoints used was variable depending on the number of involved joints but a minimum of 13, which was too numerous and too complex to try to reproduce with acupressure. We therefore opted to test the same set of acupressure points that we have previously tested in a cohort of women with breast cancer who were reporting fatigue and which resulted in improvement in both fatigue and pain [21, 22].

### Limitations and future directions

Our goal is to enable participation by a diverse patient population. We want to be as inclusive as possible, and not cause potential participants to be ineligible because of inability of travel to the cancer center. Therefore, we are conducting the trial virtually, and are providing all participants with a computer tablet. We are using video or telephone calls to ensure that participants receive and are able to operate the app, a video-based fidelity check after 2 weeks, and two additional video or phone calls to assess concomitant medications and troubleshoot any challenges that are experienced by participants. Participants will also complete patient-reported outcomes electronically via the MyDataHelps app or through an email link; those who do not have access to the internet at home will hopefully be able to access the internet at public spaces such as libraries. We will recruit patients from the cancer center clinics, but will also use outreach through social media and patient advocacy groups to try to increase the diversity of enrolled patients.

It is likely that some participants will miss completion of some questionnaires or may not completely adhere to the acupressure intervention. Study personnel will be monitoring completion of questionnaires, as outlined in the protocol. Importantly, the trial endpoints will be examined using intent to treat analyses.

Finally, the study is designed specifically to examine the effect of use of acupressure on AIMSS. As an exploratory analysis we are examining changes in the gut microbiome during the acupressure intervention, although the intervention itself was not specifically designed to target the digestive system.

## Conclusions

The trial activated to enrollment in March 2024 and is enrolling participants. Preliminarily demonstrating the feasibility and effectiveness of acupressure for treatment of AIMSS can potentially lead to a definitive, multicenter trial of acupressure for AIMSS and other cancer treatment-related toxicities. Since acupressure is self-administered, it may be more accessible to patients who are unable to use other complementary and alternative medicine approaches because of cost and availability. Ultimately improving persistence with endocrine therapy through symptom management will ideally improve disease outcomes as well as the quality of life of patients with breast cancer.

## Supporting information

S1 File Standard Protocol Items: Recommendations for Interventional Trials (SPIRIT) checklist. SPIRIT checklist for the AcuAIM clinical trial protocol.
(PDF)

S2 File AcuAIM clinical trial protocol. (PDF)
