# Supplementary material for: Self-acupressure for patients with breast cancer experiencing aromatase inhibitor-associated musculoskeletal symptoms: Protocol for the AcuAIM randomized pilot trial
Source: PLoS One. 2025 Jan 22;20(1):e0311044. doi: 10.1371/journal.pone.0311044 (PMC11753630; doi:10.1371/journal.pone.0311044)
Supplement: S2 File — (PDF) [file pone.0311044.s002.pdf]

Rogel Cancer Center

Title: Acupressure for Aromatase Inhibitor-Associated Musculoskeletal Symptoms in Patients with Breast Cancer

Protocol UMCC 2023.084

**Principal Investigator**	N. Lynn Henry, MD, PhD
**Co-Investigators**	Joan Armstrong, NP<br>Monika Burness, MD<br>Erin Cobain, MD<br>Tamara Ghormley, NP<br>Kathleen Kemmer, MD<br>Margaret Miodonski, NP<br>Aki Morikawa, MD, PhD<br>Kelly Scheu, NP<br>Anne Schott, MD<br>Jeffrey Smerage, MD, PhD<br>Catherine Van Poznak, MD<br>Megan Williams-Morad, PA-C<br>Suzanna M. Zick, ND, MPH
**Statistician(s)**	Kelley Kidwell, PhD<br>University of Michigan School of Public Health
**NCT Number** *(CT.gov)*	NCT06228768

<u>**Protocol Version Dates**</u>

Version 1: 9/29/2023

Version 2: 10/25/2023

Version 3: 11/28/2023

Version 4: 12/10/2023

Version 5: 12/17/2023

Version 6: 2/13/2024

Version 7: 2/20/2024

Version 8: 4/12/2024

Version 9: 4/17/2024

TABLE OF CONTENTS

TABLE OF CONTENTS 2
ABBREVIATIONS 4
STUDY SUMMARY 5
SCHEMA 7
1 OBJECTIVES 8
1.1 Primary Objective 8
1.2 Secondary Objectives 8
1.3 Exploratory Objectives 8
2 BACKGROUND 8
2.1 Hypothesis 8
2.2 Rationale and Background: 9
3 STUDY DESIGN 14
3.1 Description 14
3.2 Number of Patients 14
3.3 Number of Study Centers 15
3.4 Study Duration 15
3.5 Randomization 15
3.6 Recruitment 15
4 ELIGIBILITY CRITERIA 16
4.1 Inclusion Criteria 16
4.2 Exclusion Criteria 17
5 STRATIFICATION FACTORS 17
6 TREATMENT PLAN 17
6.1 Study Overview 17
6.2 Acupressure intervention 17
6.3 Randomization 18
6.4 Blinding 19
6.5 Accountability, Compliance, and Fidelity 19
6.6 Concomitant Medications and Therapies 20
6.7 Duration of Therapy 20
7 TOXICITIES AND DOSAGE MODIFICATION 20
7.1 Dose Modifications and Guidelines for Adverse Event Management 21
7.2 Supportive Care 21
7.3 Contraception 21
8 STUDY CALENDAR 22
9 STUDY PROCEDURES 24
9.1 Screening Evaluations 24
9.2 Study Plan 24
9.3 Patient-Reported Outcomes 26
9.4 Stool samples 27
10 CRITERIA FOR EVALUATION AND ENDPOINT 28
10.1 Safety 28
10.2 Stopping Rules 28
11 STATISTICAL CONSIDERATIONS 28
11.1 Statistical hypothesis 28

11.2 Sample size determination 28
11.3 Statistical Analyses 28
12 DATA SUBMISSION SCHEDULE 30
13 SPECIAL INSTRUCTIONS 30
13.1 Patient-reported questionnaires 30
14 ETHICAL AND REGULATORY CONSIDERATIONS 30
14.1 Informed consent 30
14.2 Human Subjects Protections 31
14.3 Institutional Review 31
14.4 Data and Safety Monitoring Plan 31
14.5 Adverse Events 31
14.6 Protocol Amendments 35
14.7 Protocol Deviations 35
14.8 Clinical Trials Data Bank 35
15 REFERENCES 36
16 Appendices 39
16.1 Appendix A: Patient-Reported Outcomes Questionnaires 39
16.2 Appendix B: Instructions for Collecting a Stool Sample at Home - UPS 52
16.3 Appendix C: Instructions for Collecting a Stool Sample at Home - USPS 56

ABBREVIATIONS

AI	Aromatase inhibitor
AIMSS	Aromatase inhibitor-associated musculoskeletal symptoms
BPI	Brief Pain Inventory
CTCAE	Common Terminology Criteria for Adverse Events
DSMC	Data and Safety Monitoring Committee
ER	Estrogen receptor
FM	Fibromyalgia
GRC	Global Ratings of Change
HR	Hormone receptor
ICF	Informed Consent Form
IRB	Institutional Review Board
LHRH	Luteinizing Hormone Receptor Hormone
METE	Mao Expectation of Treatment Efficacy
MICHR	Michigan Institute for Clinical and Health Research
NCI	National Cancer Institute
NSAID	Non-steroidal anti-inflammatory drugs
O-CTSU	Oncology Clinical Trial Support Unit
PARP	Poly adenosine diphosphate-ribose polymerase
PR	Progesterone receptor
SA	Sham acupressure
SAE	Serious adverse event
TA	True acupressure
TCM	Traditional Chinese Medicine

STUDY SUMMARY

Title	Acupressure for Aromatase Inhibitor-Associated Musculoskeletal Symptoms in Patients with Breast Cancer
Short Title	Acupressure-AIM
Protocol Identifier	HUM00241228
Phase	N/A
Design	Randomized, double-blind interventional trial
Study Duration	2 years
Study Center(s)	Single-center
Objectives	*Primary Objective:*<br><br>To determine the effect of 12 weeks of true acupressure versus sham acupressure on AI-associated worst joint pain, as assessed with the Brief Pain Inventory (BPI)<br><br>*Secondary Objectives:*<br><br>To determine the effect of 12 weeks of true acupressure versus sham acupressure on other AI-associated pain symptoms, including average pain and pain interference, as assessed with the BPI<br><br>To determine the effect of 12 weeks of true acupressure versus sham acupressure on other AI-associated symptoms, including anxiety, depression, insomnia, and cognitive function, as assessed with the PROMIS-29+2 Profile<br><br>To examine the adherence with 12 weeks of true acupressure versus sham acupressure in patients with breast cancer taking aromatase inhibitor therapy<br><br>*Exploratory objectives:*<br><br>To examine the association between expectation of response to acupressure as assessed with the METE and observed response based on change in BPI worst pain score<br>To examine the impact of 12 weeks of true acupressure versus sham acupressure therapy on

	the stool microbiome in patients with breast cancer taking AI therapy
Number of Subjects	25 per study arm, total 50 (goal 44 evaluable patients)
Intervention	True self-acupressure (TA)
Duration of administration	12 weeks
Reference therapy	Sham self-acupressure (SA)
Randomization	1:1, double-blind
Statistical Methodology	The primary endpoint is change in worst joint pain with 12 weeks of the intervention. Descriptive statistics including mean, standard deviation, median, interquartile range, t tests, and chi squared tests will be used to describe the patient population and the patient-reported outcomes. A linear model using generalized estimating equations and robust variance will be used with a time by group interaction to determine the difference between TA and SA. With a sample size of 44 participants (22 per arm), assuming a standard deviation of difference in worst pain score between treatment groups of 2.3 points, we will have 80% power to detect a reduction of 2 points in worst pain on the BPI at 12 weeks,[1] assuming a 5% significance level. A 2-point improvement in pain is considered a minimal clinically important difference.[1] A total of 50 participants will be enrolled to account for potential dropout, to ensure 44 participants are evaluable for the primary endpoint.

SCHEMA

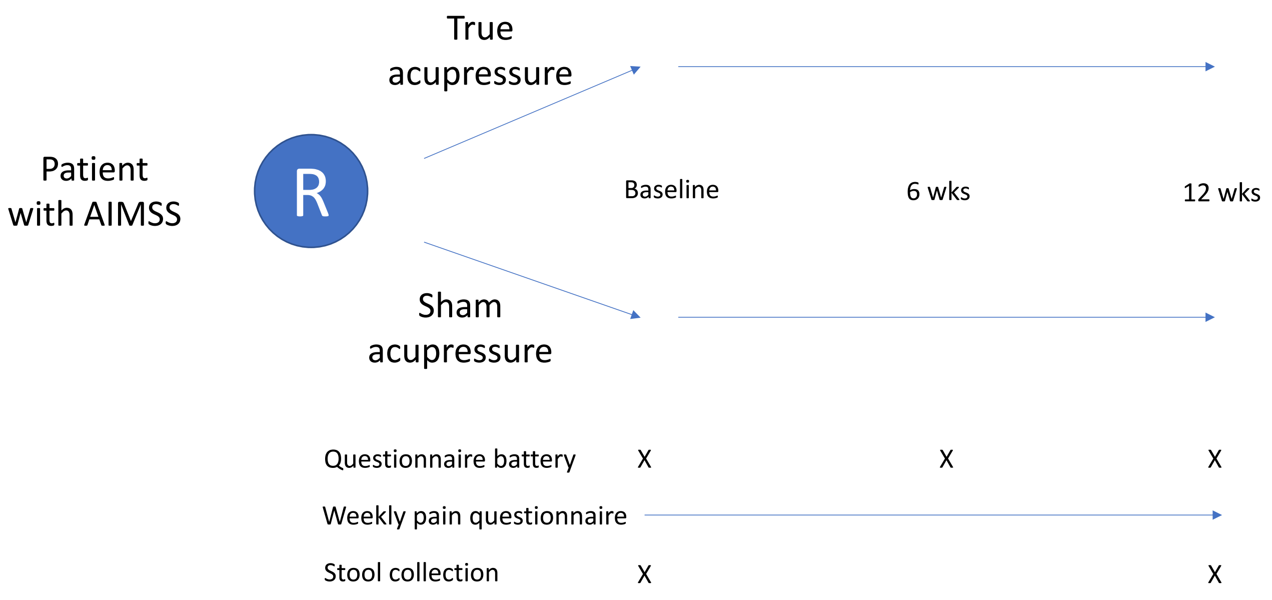

True acupressure
Patient with AIMSS
R
Baseline
6 wks
12 wks
Sham acupressure
Questionnaire battery
X
X
X
Weekly pain questionnaire
Stool collection
X
X

1 OBJECTIVES

1.1 Primary Objective

To determine the effect of 12 weeks of true acupuncture versus sham acupuncture on AI-associated worst joint pain, as assessed with the Brief Pain Inventory (BPI).
Endpoint: Change in worst joint pain with 12 weeks of the intervention

1.2 Secondary Objectives

1. To determine the effect of 12 weeks of true acupuncture versus sham acupuncture on other AI-associated pain symptoms, including average pain and pain interference, as assessed with the BPI.
   Endpoint: Change in (a) average joint pain and (b) pain interference with 12 weeks of the intervention

2. To determine the effect of 12 weeks of true acupuncture versus sham acupuncture on other AI-associated symptoms, including anxiety, depression, insomnia, and cognitive function, as assessed with the PROMIS-29+2 Profile.
   Endpoint: Change in (a) anxiety, (b) depression, (c) sleep disturbance, (d) fatigue, and (e) cognitive function with 12 weeks of the intervention

3. To examine the adherence with 12 weeks of true acupuncture versus sham acupuncture in patients with breast cancer taking aromatase inhibitor therapy.
   Endpoint: Number of days acupressure is performed during the 12 weeks of the intervention

1.3 Exploratory Objectives

1. To examine the association between expectation of response to acupressure as assessed with the METE and observed response based on change in BPI worst pain score
   Endpoint: Expectation of response to acupressure based on the METE questionnaire

2. To examine the impact of 12 weeks of true acupuncture versus sham acupuncture on stool microbiome in patients with breast cancer taking aromatase inhibitor therapy.
   Endpoint: Change in (a) bacterial phyla, (b) bacterial genera, and (c) Shannon diversity index with 12 weeks of the intervention

2 BACKGROUND

2.1 Hypothesis

We hypothesize that 12 weeks of true acupuncture will result in greater improvement in joint pain compared to 12 weeks of sham acupuncture.

2.2 Rationale and Background:

Aromatase inhibitor (AI) therapy for breast cancer

Breast cancer is the most common cancer diagnosis among women in the United States, affecting one in eight women in her lifetime. With advancements in screening and treatment, nearly 90% of women diagnosed with breast cancer in the United States survive beyond five years and comprise an estimated 25% of cancer survivors. About 80% of breast cancers diagnosed in postmenopausal women are hormone receptor positive (HR+). For these women, daily oral adjuvant endocrine therapy for five to ten years following primary treatment (surgery, radiation, chemotherapy) is indicated.[2]

Third generation AIs are the preferred endocrine treatment for postmenopausal women with early-stage HR+ breast cancer. Aromatase is the key enzyme that converts androgens to estrogens. In postmenopausal women, aromatase is only expressed in non-glandular tissues including fat, liver, brain, and breast tissues.[3] AIs prevent the biosynthesis of estrogen in these tissues, thus preventing the proliferation of HR+ breast cancer cells. Large-scale comparative effectiveness trials demonstrated the superiority of AIs over other endocrine treatment (i.e., tamoxifen), in terms of prolonged disease-free survival, rates of distant metastasis, and contralateral breast cancer, as well as a preferable toxicity profile.[4]

AI-Associated Musculoskeletal Symptoms (AIMSS)

Despite their clinical utility, AIMSS affect ~50% of the 200,000 patients who start AI therapy per year in the US.[5] Musculoskeletal symptoms, including arthralgias, myalgias, and joint stiffness, are the primary symptoms reported by AI-treated patients. These symptoms typically emerge within about 3 months of AI initiation, and peak at 6 months.[5, 6] Despite considerable research, the etiology of AIMSS remains poorly understood.[5, 7-10] Postulated mechanisms include inflammation and reductions in naturally anti-nociceptive properties of estrogen.[11, 12] While multiple randomized clinical trials of AIMSS treatments have been conducted, only a few interventions including acupuncture and duloxetine have demonstrated improvement in musculoskeletal pain and stiffness,[13, 14] and are currently unavailable to most patients due to access issues (acupuncture) and perceived stigma and toxicity (duloxetine).

AIMSS can lead to early treatment discontinuation in one-fifth of patients, which can increase risk of breast cancer recurrence by 45-50%.[5, 15-17] Despite well-established clinical benefits of long-term AI treatment, more than 20% of patients discontinue AI therapy prematurely, primarily because of toxicity of therapy. While the primary toxicity of therapy is AIMSS, AI-treated women also report bothersome vasomotor and gynecologic symptoms, as well as insomnia, fatigue, anxiety, and depression. In addition, studies have shown an increase in inflammatory markers in patients with co-existing symptoms, supporting an underlying inflammatory mechanism.[12] Discontinuation of AI therapy before the recommended 5-10 years has been shown to increase risk of cancer

recurrence and mortality.[15-17] Therefore, well-tolerated and effective interventions to improve AI-associated symptoms, primarily AIMSS, are urgently needed.

Acupuncture and AIMSS

Acupuncture is a traditional Chinese therapy that involves insertion of fine, single-use, sterile needles into acupoints throughout the body. It is a nonpharmacologic modality used for treating a variety of conditions, including pain. A multicenter randomized, controlled trial of true acupuncture, sham acupuncture, and wait-list control was conducted by SWOG.[14] In the trial, participants randomized to true acupuncture received treatment at standard acupoints, and could also receive treatment to up to 3 additional acupoints depending on sites of pain.[18] From baseline to 6 weeks, the mean worst pain score decreased by 2.05 points in the true acupuncture group, by 1.07 points in the sham acupuncture group, and by 0.99 points in the wait-list control group. Between the true and sham acupuncture groups, the adjusted difference was 0.92 points (95% CI 0.20-1.65, p=.01). Based on these results, acupuncture is an effective therapy for treating AIMSS. However, uptake is incomplete because of the cost of acupuncture, limited availability of acupuncturists, and for some patients, discomfort with needles.

Acupressure

Acupressure is a technique derived from acupuncture. It is a component of Traditional Chinese Medicine (TCM) in which pressure is applied to specific acupoints on the body using a finger or small device to address health issues, rather than stimulating the acupoints using needles. Acupressure has shown promise for treating fatigue and other symptoms in cancer patients and survivors.[16-20] It can be delivered by a practitioner or can be self-administered.

Zick and colleagues performed a randomized clinical trial of relaxing and stimulating self-acupressure in fatigued breast cancer survivors.[19] In this study, 288 participants were randomized to one of three groups: relaxing acupressure (n=94); stimulating acupressure (n=90); or usual care (n=86). Participants performed acupressure for 12 weeks between 3 to 14 times per week depending on group. At 6 weeks, 66% of participants receiving relaxing acupressure, 61% receiving stimulating acupressure, and 31% receiving usual care reported normal levels of fatigue. Improvements in fatigue persisted over the next 10 weeks in both acupressure arms.

Self-administered Relaxation Acupressure Decreases Chronic Pain

“Relaxation acupressure” is used to describe a set of acupressure points that are derived from a TCM formula designed to improve insomnia and pain. This combination of acupoints is also what we have found to reduce fatigue and pain symptoms in cancer survivors.[20, 21] We examined the effect of 6 weeks of self-administered relaxation acupressure on chronic pain in breast cancer survivors with fatigue. In breast cancer survivors with fatigue, we identified a significant decrease in pain severity and

interference at both the 6 and 10 week time points in the relaxation acupressure group compared to the usual care group (Table 1).[22] We compared the relaxation acupressure formula to usual care (n=89: n=46 relaxation acupressure and n=43 usual care) in breast cancer survivors with a history of stage 0-IIIA disease who were experiencing chronic pain (pain for at least 6 months, with pain on most days reported as ≥ 3 on a pain visual analog scale). Women had completed all cancer-related treatments at least 12 months before enrollment apart from hormone therapies. We excluded women who were currently taking aromatase inhibitors. Women had to have been on stable chronic pain medications for at least 3 months prior to entry into the study and have no planned addition or change of dose of medications. The Brief Pain Inventory (BPI) severity and interference subscales were used to determine changes in pain. Independent sample t-tests were performed on mean differences of changes in pain scales through time (end of treatment = 6 weeks and one month washout = 10 weeks) by group. Clinically relevant changes in pain were defined as a 30% relative reduction or a 2-point decline in BPI pain severity or interference between baseline and 6 weeks.

Mean age was 59.0 ± 7.2, 100% of the women were Caucasian and 53% were post-menopausal. There were no significant differences in chronic pain medications (e.g., NSAIDs, opioids, acetaminophen, anticonvulsants, antidepressants) between groups. Changes in pain and percentage of participants who experienced clinically significant changes are shown below in Table 1 and Figure 1. Adherence to performing acupressure treatments as recorded in logbooks was 79.8±22.9% (34 out of 42 total treatments), and 53 out of 74 women (72%) completed at least 70% of their acupressure treatments. Fidelity was 89.2±14.4% half-way through the acupressure intervention (at week 3), and had improved to 94.6±10.2% at week 6.

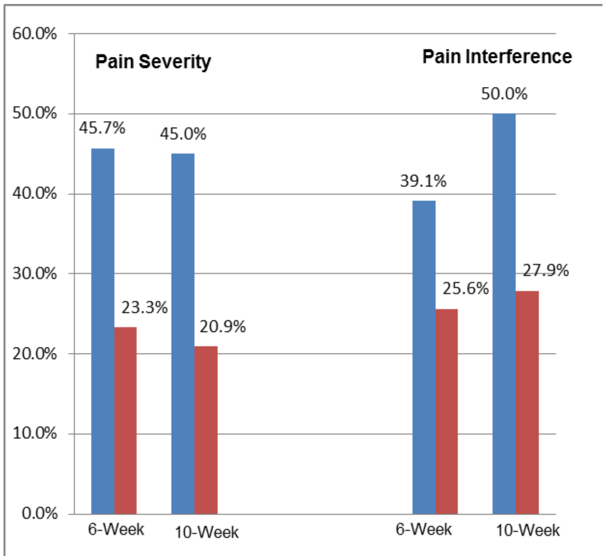

Figure 1. Percent of participants who had a clinically significant reduction in self-reported pain (Blue: relaxation acupressure, Red: usual care)

Table 1 Change In Brief Pain Inventory (BPI) Pain Severity and Interference			
**BPI Pain <u>Severity</u>**	**Relaxation Acupressure N=46**	**Usual Care N=43**	**P-value***
	Mean ± STD	Mean ± STD	
Baseline Pain Severity	4.8 ± 1.6	4.6 ± 1.8	0.51
Week 6 Pain Severity	3.5 ± 2.2	4.6 ± 2.1	0.02
Week 10 Pain Severity	3.8 ± 2.2	4.6 ± 2.2	0.10
Point Change at 6 Weeks	-1.3 ± 2.0	0.1 ± 2.0	0.003
Point Change at 10 Weeks	-1.0 ± 1.8	0.02 ± 1.6	0.006
Percentage Change at 6 Weeks	-27.2 ± 48.8	20.2 ± 122.3	0.002
Percentage Change at 10 Weeks	-20.3 ± 41.9	6.6 ± 52.3	0.01
**BPI Pain <u>Interference</u>**	**Relaxation Acupressure N=46**	**Usual Care N=43**	**P-value**
	Mean ± STD	Mean ± STD	
Baseline Pain Interference	3.7 ± 2.1	3.8 ± 2.4	0.87
Week 6 Pain Interference	2.6 ± 1.8	4.1 ± 2.4	0.002
Week 10 Pain Interference	2.6 ± 2.3	3.7 ± 2.3	0.03
Point Change at 6 Weeks	-1.1 ± 2.2	0.2 ± 2.0	0.003
Point Change at 10 Weeks	-1.1 ± 1.6	-0.1 ± 1.5	0.004
Percentage Change at 6 Weeks	-15.0 ± 80.6	22.8 ± 127.2	0.10
Percentage Change at 10 Weeks	-27.6 ± 55.3	2.8 ± 66.8	0.02
***Independent sample t-test**			

Self-acupressure

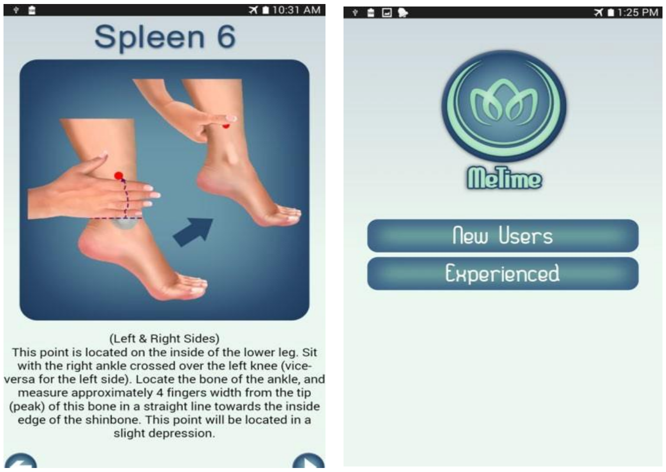

Figure 2. Sample MeTime Acupressure Screen

An advantage of self-acupressure is that once a person learns the technique, they are free to control the frequency and dose of the intervention. Since the intervention can be easily learned (generally in less than 15 minutes),[24] we reasoned that an online or mobile application (app) could increase its utilization. In a recent pilot survey of cancer patients with fatigue, use of technology such as a computer program or smartphone app was the most popular response for learning acupressure. Consequently, we developed a mobile app in association with focus groups consisting of cancer survivors (six focus groups consisting each of eight to ten women) and the

University of Michigan 3D Media Laboratory. **The acupressure app is called MeTime Acupressure** (Figure 2). One concern with some app-based interventions is that the company that initially develops the app may cease to make it available or support it. **Our app was developed at the University of Michigan by the Principal Investigator** and thus there is no chance it will not be available in the future. Importantly, further refinements of the app can be done in an iterative manner.

Focus group participants also asked for a device to accompany the app to learn and apply an accurate amount of pressure to each acupoint (the hardest skill to learn for participants and one they expressed as being of high importance).[23] They also wanted a device that was comfortable to use and could minimize any strain in the hand or fingers from applying the acupressure. Having a device that is easy to hold may be particularly important for patients with musculoskeletal symptoms that include joint pain and stiffness and decreased grip strength. To achieve this goal, we partnered with Arbor Medical Innovations, a Michigan-based technology company to translate their expertise in handheld devices to create the "AcuWand" device. For this study, we are going to provide participants with the shell of the AcuWand because it incorporates an ergonomic handle so the user can easily grasp the device and apply pressure through a sprung probe with a soft rubber tip. **The AcuWand was designed in collaboration with users and can objectively and comfortably enable breast cancer survivors to deliver pressure to each acupressure point.**

Assessment of AIMSS

No published scales have been established or validated to specifically measure the effects of treatments for AI-induced joint pain and stiffness. Therefore, we have chosen to use a combination of scales to assess the effect of acupressure for improving tolerance of AI therapy. As has been used in multiple prior trials of treatments for AIMSS, patients will complete self-reported questionnaires including the BPI-Short Form[23] to assess severity of joint pain and the Global Ratings of Change (GRC) scale to identify overall change in pain/stiffness since starting AI therapy. To assess other symptoms related to endocrine therapy, patients will complete the PROMIS-29+2.[24] To assess expectation of benefit from acupressure, patients will complete the Mao Expectation of Treatment Efficacy (METE).[25] Finally, at baseline patients will complete the 2011 Fibromyalgia (FM) Survey to assess nociplastic pain. We will also collect data on frequency of administration of acupressure, use of concomitant medications, and whether participants believe they were randomized to the true or sham acupressure study arm. All of these questionnaires are described in greater detail in section 10.

Mechanism underlying AIMSS Development

As noted above, despite considerable research the etiology of AIMSS remains uncertain.[5, 7, 8] Postulated mechanisms include inflammation and reductions in naturally anti-

nociceptive properties of estrogen, although the actual mechanism remains unclear. Researchers have investigated associations between both clinical factors and genetic and biochemical biomarkers and development of musculoskeletal symptoms.[5, 26] In particular, we have demonstrated that pre-existing nociplastic pain and greater increase in pain sensitivity with AI therapy are associated with shorter time to AI discontinuation because of symptoms.[7, 27] However, we have been unable to identify associations between systemic inflammatory cytokine concentrations and development of AIMSS.[8] To date it has been challenging to elucidate the etiology of AIMSS or to identify effective management options.

The gut microbiota plays a critical role in human health, including metabolism, obesity, and joint injury.[28] Disruption of the gut microbiota is influenced by many factors, including diet and medications, and has been closely linked to the development of chronic inflammation.[29] Studies have shown that the gut microbiome is influenced by estrogen, and also influences estrogen levels.[30] To date, there have been few studies of the impact of estrogen deprivation on the gut microbiome, or its subsequent effects. In a mouse model of polycystic ovarian syndrome, AI-treated mice had a reduction in fecal microbial richness and alpha diversity over time, including increases in bacterial species associated with inflammation.[31] However, it is unknown either how estrogen deprivation influences the microbiome or whether there are associations between changes in the microbiome and development of treatment-emergent toxicity. Also, acupressure and acupuncture have been associated with an increased abundance of anti-inflammatory bacteria in the gut,[32, 33] Therefore, during conduct of this study we will request that participants submit stool samples so that we can determine whether there are changes in the gut microbiome that occur during treatment with acupressure, and explore whether the changes are associated with reduction in AIMSS symptoms.

3 STUDY DESIGN

3.1 Description

This will be a randomized, double-blinded trial of true versus sham self-acupressure to examine the effect of treatment on AIMSS in postmenopausal women with breast cancer. We will also examine effects of acupressure on other symptoms that commonly develop in AI-treated patients. In addition, change in stool microbiome with acupressure treatment will be examined in an exploratory way to obtain preliminary information about the effect of acupressure on the gut microbiome in this condition.

3.2 Number of Patients

A total of 50 patients will be enrolled, assuming a 10% dropout rate, for a total of 44 evaluable patients. Patients who discontinue trial participation prior to initiating study-directed therapy will be replaced.

3.3 Number of Study Centers

This will be a single center trial conducted at the Rogel Cancer Center at the University of Michigan.

3.4 Study Duration

The study is anticipated to be open for 2 years.

3.5 Randomization

Participants will be randomized 1:1 to true versus sham acupressure. Both clinicians and participants will be blinded to study arm.

3.6 Recruitment

We may use the following sources for recruitment of participants:

- *Social Media Advertising.* We will work with the Michigan Institute for Clinical and Health Research (MICHR) and the Cancer Center Department of Communications to provide social media advertisement support. This may include Facebook, X (i.e., Twitter), Instagram and Search Engine Marketing via Google search terms. We will also utilize an organic social media recruitment approach, directly contacting support groups and other applicable online groups to reach our intended audience. We will request that our approved marketing materials be shared with these groups. Last, we may choose to create social media accounts/groups managed and monitored by the study team. Content on these accounts that is intended for participant recruitment will be selected from the approved social media kit. We may also choose to share news, articles, and webinar information that is otherwise widely available to the public. We will not accept or share PHI through social media accounts and will direct all communications to our study team email.

- *U-M Rogel Cancer Center Social Media and Health Communications Core.* We may choose to use the images and texts in the MICHR advertisements to advertise using these platforms.

- *University of Michigan Cancer Registry.* We may request a listing of breast cancer survivors that are likely eligible for the study, i.e., diagnosed within the eligible time frame. Study team members will send letters, emails, or texts as appropriate. We will merge data using the medical record number to obtain email and phone numbers from Data Direct.

- *University of Michigan Rogel Cancer Center.* We will let patients who are currently being treated for breast cancer at the cancer center and who are experiencing AIMSS know about the clinical trial, in case they are interested in participating if they are found to be eligible.

- *Breast Cancer Patient Advocate and Non-Profit Groups.* We may reach out to groups that advocate for patients with breast cancer to help advertise to their members.

- *UMHealthResearch.org and ResearchMatch.org.* UMHealthResearch is a secure tool that facilitates a partnership between health researchers and volunteers. We will post our study here for potential participants to learn more about the study and reach out to study personnel if they are interested in participating. Like UMHealthResearch, Research Match is a participant recruitment and feasibility analysis tool for researchers. We will post our study information here as well for potential participants to learn more and to contact us if they wish to consider study participation.

- *Snowballing.* We may choose to email participants who have completed the study and share our flyer and social media content with them. If they would like to, they may choose to share our study information to their own personal social media accounts or groups that they may be members of. This is considered a "word of mouth" recruitment technique.

4 ELIGIBILITY CRITERIA

4.1 Inclusion Criteria

Yes/No (Response of "no" = patient ineligible)

4.1.1 _____ Female or male subject aged ≥ 18 years

4.1.2 _____ Taking the <u>currently prescribed</u> aromatase inhibitor therapy (anastrozole, exemestane, or letrozole) for adjuvant or palliative treatment of breast cancer or for chemoprevention for at least 3 weeks and no more than 2 years at the time of enrollment.

4.1.3 _____ Planning to take the same AI therapy for at least 12 weeks.

4.1.4 _____ New or worsening joint pain and/or myalgias since starting the AI therapy, with *worst* pain score of at least 4 out of 10 on the BPI over the 7 days prior to enrollment.

4.1.5 _____ Completion of radiation therapy, if given, for treatment of breast cancer.

4.1.6 _____ Completion of chemotherapy, if given. Concurrent use of LHRHa therapy, anti-HER2 therapy, bisphosphonate therapy, PARP inhibitor therapy, and CDK4/6 inhibitor therapy is permitted.

4.1.7 _____ Patients receiving treatment with NSAIDs, acetaminophen, opioids, duloxetine, cannabinoids, gabapentin, and/or pregabalin must have been taking a stable dose for at least 30 days prior to enrollment if they plan to continue the drug during study participation. If they do not plan to take the medication during study participation, they should stop the medication at least 7 days before initiation of study treatment.

4.1.8 _____ Able to read, understand, and self-complete questionnaires in English.

4.1.9 _____ Able to access WiFi/internet and willing to use an email account or download and use the MyDataHelps app.

4.1.10 _____ Able to provide informed consent and willing to sign an approved consent form that conforms to federal and institutional guidelines.

4.2 Exclusion Criteria

Yes/No (Response of “yes” = patient ineligible)

4.2.1 _____ Use of acupuncture or acupressure in the past year, or planned use of acupuncture during study participation.

4.2.2 _____ Use of systemic or transdermal estrogen during study participation.

4.2.3 _____ Planned surgery during the 12-week study period.

4.2.4 _____ Concurrent medical or arthritis disease such as painful bone metastases or active rheumatoid arthritis or inflammatory arthritis that could confound or interfere with evaluation of pain or efficacy. Patients with osteoarthritis and asymptomatic bone metastases are eligible.

4.2.5 _____ Patients with a prior or concurrent malignancy whose natural history or treatment, in the opinion of the treating investigator, has the potential to interfere with the safety or efficacy assessment of the investigational regimen.

5 STRATIFICATION FACTORS

None

6 TREATMENT PLAN

6.1 Study Overview

Participants with AIMSS will be randomized 1:1 to either true or sham self-acupressure interventions. Participants will use the provided modified MeTime Acupressure mobile app and AcuWand to self-administer acupressure daily for 12 weeks. All participants will complete weekly pain questionnaires, every 6-week symptom assessment batteries, and will be asked to provide optional stool samples for microbiome assessment prior to study entry and at 12 weeks. Participants randomized to the sham treatment will have the option of receiving the app with the true acupressure points after completion of the 12 week study intervention.

6.2 Acupressure intervention

Self-acupressure interventions (true and sham) will be delivered using our modified MeTime Acupressure mobile app. The MeTime Acupressure app has been renamed “Study App” and the icon changed so participants will not be able to locate it in the Apple or Google Play Stores and unblind

themselves. The sham and true acupressure apps are identical in all aspects except for the acupoint locations. The acupressure app will be loaded onto computer tablets by our computer information technology (IT) support person and provided to participants. Participants will also receive an AcuWand to be used in association with the acupressure app to help apply pressure to acupoints. The participants will receive directions within the acupressure app on how to use the AcuWand. Of note: our IT support person will be available to help participants who are unfamiliar with or have any issues with their tablet or the app.

True Acupressure. The true acupoints in this trial are identical to the relaxing acupressure acupoints that have been studied in prior trials, as described in the background section. There are 5 acupoints with 4 of the acupoints performed on both the left and right sides of the body (total of 9 points to stimulate). Each of the 9 acupoints will be stimulated for 3 minutes per point with the AcuWand giving a total treatment time of 27 minutes daily. The relaxation acupoints are:

- *Yin tang* (Unilaterally): forehead, between eyebrows
- *Anmian* (EX17) (Bilaterally): posterior aspect of the neck
- Heart 7 (HT7) (Bilaterally): palmer surface of the hands on the wrist crease
- Spleen 6 (SP6) (Right and Left/bilaterally): inside of the lower leg
- Liver 3 (LV3) (Bilaterally): foot

The true points were selected based on existing data showing their effectiveness for treating pain and fatigue.[21,22]

Sham Acupressure. Sham acupoints were selected in locations where no known acupoints exist. There are 5 acupoints with 4 of the acupoints performed on both the left and right sides of the body (total of 9 points to stimulate). None of these points are on meridians nor are they near actual points (at least one inch away from meridians and points). They were chosen to be in the same general body quadrant as the relaxation points. Each of the 9 acupoints will be stimulated for 3 minutes per point giving a total treatment time of 27 minutes daily. The sham acupoints are:

- Pressure Point #1 (Unilaterally): This point is located on the side of the head, above the right ear.
- Pressure Point #2 (Bilaterally): This point is located on the muscle of the upper arm. Located in the middle point directly between the crease of the elbow (when a[illegible]exed) and the shoulder muscle one finger width toward the biceps muscle.
- Pressure Point #3 (Bilaterally): This point is located on the upper thigh muscle on the lateral side of the leg.
- Pressure Point #4 (Bilaterally): This point is located on the lower thigh muscle on the lateral side of the upper leg.
- Pressure Point #5 (Bilaterally): This point is located on the lateral side of the lower leg and is located midway between the line connecting the outer ankle bone and the knee.

6.3 Randomization

The biostatistician will provide randomization lists for the study. Participants will be randomized to one of the two study arms in a 1:1 ratio: True acupressure or Sham acupressure. A random generator for block randomization in blocks of 2 or 4 participants will be used.

6.4 Blinding

Our IT support person will place the computer tablets—already loaded with either the true or sham acupressure app - and the AcuWands into opaque shipping boxes per our randomization code (i.e., a tablet with the sham acupressure app loaded on it would be placed in a shipping box labeled with a study ID assigned to the sham arm). The outside of the shipping boxes will be labeled with study IDs. This will ensure that only our IT support person and our staff member who is doing the fidelity assessments will be unblinded, but all other study staff and researchers will remain blinded while participants are participating in the 12-week intervention. The IT support person will not be responsible for collecting or analyzing any study data. Participants in the two self-acupressure arms will be blinded as to which acupressure treatment they have been randomized until the 12-week study intervention is complete.

6.5 Accountability, Compliance, Fidelity, and Debriefing

Adherence. Adherence to acupressure will be assessed by asking participants to record on an electronic diary if and when they performed acupressure, the length of time they spent performing acupressure, and whether they took any breaks during treatment. Bi-weekly emails will be used to troubleshoot and encourage adherence. Since this is an intent to treat analysis, failure to complete the diary will not be considered a deviation and will not have to be reported to the IRB.

Testing Participant Fidelity. As detailed in our prior study,[26] assessment of fidelity is done by research staff during week 2 (+/- 1 week) using a Zoom call (we will install Zoom on the computer tablets and assist participants in using this program as needed). Participants will be asked to identify the location, stimulation technique, and the amount of pressure (be able to demonstrate correct use of their AcuWand) of their acupoints. The number of acupoints correctly located will be recorded as will the adequacy of stimulating the acupoints (recorded as "yes" or "no"). The percentage of correct answers 0 to 100% will be calculated, based on circular (clockwise or counterclockwise) motion, maintaining contact with skin, and applying pressure such that the nail of the applying finger/thumb blanches. The research staff member who assesses fidelity will be unblinded to treatment arm and will not be involved in assessment of symptoms.

Debriefing. Once participants complete study participation they will be sent the debriefing document by email which notifies them that one of the arms of the study contained true acupoints whereas the other arm contained sham acupoints that were not thought to be effective. They will be offered the option of being given the other version of the app than the one they were randomized to. If they wish to receive the app, then we will work with the IT representative on the study team to send them the alternative version of the app.

6.6 Concomitant Medications and Therapies

6.6.1 Allowed Therapy

Patients are permitted to take concomitant aromatase inhibitor therapy, LHRH agonist therapy, anti-HER2 directed therapy, CDK4/6 inhibitor therapy, and/or anti-osteoclast therapy. Patients are permitted to use vaginal estrogen preparations.

Patients who are taking opioids, NSAIDs, acetaminophen, duloxetine, cannabinoids, gabapentin, and/or pregabalin at the time of study drug initiation should continue to take the same dose of medication for the 12-week study duration.

6.6.2 Prohibited Therapy

Patients must avoid taking systemic or transdermal estrogen products (vaginal estrogen preparations are permitted).

Participants must not undergo acupuncture during study participation.

6.7 Duration of Therapy

Patients will be treated with self-acupressure for 12 weeks (+/- 2 days).

6.7.1 Criteria for discontinuation of treatment ("off treatment")

The following will result in study treatment discontinuation:

- Discontinuation of AI therapy, defined as:
    - Discontinuation of the currently prescribed AI therapy for more than 7 days
- Evidence of new cancer, cancer recurrence, or cancer progression
- Unacceptable toxicity (see section 8.1)
- Delay of 14 consecutive days of study treatment (acupressure) due to any reason
- Initiation of acupuncture
- The participant may discontinue study treatment (acupressure) at any time for any reason including participant request to be withdrawn from study
- Death

7 TOXICITIES AND DOSAGE MODIFICATION

This study will utilize the CTCAE (NCI Common Terminology Criteria for Adverse Events) Version 5.0 (https://ctep.cancer.gov/protocoldevelopment/electronic_applications/docs/CTCAE_v5_Quick_Reference_5x7.pdf) for adverse event and serious adverse event reporting.

7.1 Dose Modifications and Guidelines for Adverse Event Management

There will be no dose modifications for this intervention.

7.2 Supportive Care

All supportive measures consistent with optimal patient care may be given throughout the study except for acupressure or acupuncture.

7.3 Contraception

Since this is a clinical trial of a non-pharmacologic agent that is only used for treatment of men or postmenopausal women, no methods to avoid contraception are mandated.

8 STUDY CALENDAR

Required Studies	Screening phone call [a]	Baseline Week 0 [b]	Treatment weeks 1-5 (+/- 2 days) [c]	Treatment week 6 (+/- 2 days) [c]	Treatment weeks 7-11 (+/- 2 days) [c]	End of treatment week 12 (+/- 2 days) [c,d]
**Screening survey**	X					
**Informed consent**	X					
**Patient info and demographics**	X					
**Cancer history** [e]		X				
**Concomitant medications** [f]		X		X		X
**Study phone calls** [f]		X		X		X
**Study emails** [m]			X	X	X	
**Patient reported questionnaires** [g]						
- **Brief Pain Inventory**	X [h]	X	X [h]	X	X [h]	X
- **PROMIS Profile 29**		X		X		X
- **GRC**				X		X
- **METE**		X				
- **FM Survey**		X				
- **MASS**				X		
- **Post-treatment survey**						X
**Stool collection for microbiome** [i]		X				X
**AI therapy (standard of care)**		→	→	→	→	→
**Acupressure/AI diary** [k]		→	→	→	→	→
**Intervention fidelity assessment**			X [j]			
**Debriefing**						X [l]

a. All screening procedures can take place remotely, via phone, text, or email. The informed consent can be completed remotely using SignNow or other IRB-approved platform, or can be completed in person.
b. Baseline visit can take place up to 4 weeks after the screening visit and will take place remotely. The study coordinator will contact the participant and conduct the baseline visit via phone or Zoom once the participant has received the device and AcuWand. The patient should start the study intervention within 2 days of the baseline visit.
c. Timed based on baseline visit.
d. As per section 6.7.1, if participants discontinue AI and/or study intervention they should continue to complete patient-reported questionnaires as originally planned, timed based on baseline visit. If they have been off AI and/or study intervention by more than 4 weeks at the time of 12 week study assessment, stool collection should not take place.
e. Participants will be to self-report their cancer medical history (date of diagnosis, disease stage, surgery type and date, chemotherapy type and dates if given, radiation dates if given, and names and approximate dates of all prior endocrine therapy). Participants will also be asked to submit copies of medical records if possible to verify the information.
f. Study phone calls will be performed at baseline and weeks 6 and 12. At baseline visit, study coordinator will confirm that the participant has received the tablet and AcuWand, and answer any questions that have arisen. At weeks 6 and 12, study coordinator will assess adverse events. At all visits, study coordinator will assess concomitant medications (including pain medications, antidepressants, anxiolytics, sleep medication, and use of AI therapy) based on participant self-report.
g. All patient-reported questionnaires will be sent to patient electronically. For baseline, week 6, and week 12, questionnaires will be completed electronically within 2 days before or after the study coordinator phone call. It is preferable to have patients complete the questionnaires before each phone call. Texts, emails, or phone calls will be used to remind patients to complete questionnaires. If a scheduled phone call is rescheduled after the questionnaires have been completed by the patient, they will not be considered out of window. If a patient declines to complete questionnaires electronically or if the patient has not completed them within 2 days after the visit, they will be completed by phone.

h. Worst and average pain questions only. At screening, only worst pain will be assessed.
i. Stool samples will be collected prior to initiation of acupressure and during week 12. Patients will be provided with kits to submit samples by mail. If participants have been off AI and/or study intervention by more than 4 weeks at the time of 12 week study assessment, stool collection should not take place.
j. Fidelity assessment will be performed week 2 (+/- 1 week) by a study team member who is not blinded to study arm.
k. Acupressure/AI diary will be sent electronically daily via the MyDataHelps app. Participants will record details regarding acupressure therapy, as well as whether or not they took the AI medication.
1. Once participants have completed the study, they will be debriefed and offered an opportunity to receive a copy of the results. All participants can opt to receive the other version of the app.
m. Bi-weekly emails will be sent to participants to troubleshoot issues and encourage adherence (as mentioned in section 6.5).

9 STUDY PROCEDURES

9.1 Screening Evaluations

All potential participants will be pre-screened by phone, email, text, or in person using a basic questionnaire to confirm their potential eligibility to potentially participate. A review of inclusion/exclusion criteria will be conducted to determine the patient's eligibility for enrollment. In addition, a single item from the *Brief Pain Inventory (below; BPI)* will be used to determine baseline worst pain. These measures will be collected before consent and enrollment into the study and will therefore be kept to the absolute minimum to determine eligibility. Study procedures will be reviewed with the patient, and documentation of informed consent will be obtained. After signing the informed consent form on paper or electronically using MyDataHelps, SignNow, or other program approved by the UM IRBMED, patients will be assigned a unique study ID number in sequential order. The participant's email address and phone number will be recorded. Medical records will be obtained, when possible, to confirm eligibility.

The following medical history elements will be obtained from the patient and, when possible, confirmed in the medical record: date of breast cancer diagnosis, tumor information (e.g., tumor histology, receptor status), treatment information (e.g., surgery type and dates, chemotherapy doses and dates, radiation therapy doses and dates), and information about other relevant medical co-morbidities (as outlined on the case report form).

9.2 Study Plan

Study related screening procedures can only begin once the patient has signed a consent form.

Patients must meet all of the eligibility requirements listed in Section 4 prior to registration. All eligibility will be reviewed and confirmed by PI or co-I or their delegate prior to enrollment.

Patients must be registered before receiving any study treatment and must begin study intervention within 4 weeks of registration. See study calendar (section 8) for details.

9.2.1 Baseline

- Baseline assessment should be conducted within 28 days of enrollment. Baseline assessment should occur after the participant signs informed consent and is determined to be eligible, and prior to initiation of study intervention.
- Patients will have a brief virtual study visit or phone call with a study coordinator to review the study intervention app, use of the AcuWand, and the MyDataHelps app/website, and to assess baseline concomitant medications.
- Patients will complete questionnaires online using MyDataHelps prior to initiation of study intervention. If questionnaires have not been completed at the time of the study visit described above, the study coordinator will ensure that the patient has created an account and received the notification during that study visit. If the questionnaires are not completed by 2 days after the study visit, the study coordinator will contact the patient to complete the questionnaires by phone, and to confirm when the patient is starting the study intervention.
- Optional stool sample collection for microbiome assessment in patients willing to collect samples. Samples should be collected before starting the study intervention. If a patient does not collect a sample, or does not submit it for analysis, it will not be considered a deviation.

9.2.2 Evaluation during study participation

- Daily, participants will complete an electronic study diary to report use of the study intervention and AI therapy.
- Weekly, participants will be asked to complete two questions either electronically or by phone about worst and average pain severity.
- At 6 and 12 weeks (+/- 2 days), participants will undergo assessment with a battery of electronic questionnaires. If a patient is unable to complete them electronically, they will be asked by phone.
- At 6 and 12 weeks (+/- 2 days), participants will have a virtual study visit with a study coordinator (phone or video) to review concomitant medications and adverse events. If a patient is unable to complete a visit by phone or video, they will be asked by email.
- During week 12 (or at off study if a participant discontinues study participation early), participants who submitted optional baseline stool samples will be asked to undergo repeat optional stool sample collection. If a participant does not collect a sample, or does not submit it for analysis, it will not be considered a deviation.

9.3 Patient-Reported Outcomes

Administering Study Assessments. Each participant will be instructed to download the MyDataHelps app on their personal smartphone or can use the web version of MyDataHelps on the study-provided tablet. They will be required to create an account on MyDataHelps. Enrolled participants with the app on their smartphone will receive notifications, emails, or texts to complete study measures per protocol.

For those who are not using the MyDataHelps app on their smartphone or tablet, notifications to complete questionnaires will be sent via email or text. E-mail is required for participants who are unable to use the MyDataHelps app on their smartphone and who do not have access to text notifications, so that they know to complete surveys on the MyDataHelps website. Participants who need to receive email notifications about available surveys but who do not have an email account will be assisted in obtaining a free Gmail account and how to access it on their computer tablet by study staff.

The validated instruments used in this study are as follows (See Appendix A):

Brief Pain Inventory (BPI): The BPI is a 17-item patient self-rating scale that assesses sensory and reactive components of pain.[23] For sensory components, it addresses severity, location, chronicity, and degree of relief due to therapy. For reactive components, it assesses depression, suffering, and perceived availability of relief. Reliability has been demonstrated over short intervals using test retest item correlation; worst pain, r=0.93, usual pain, r=0.78, pain now r=0.59. It has been validated in patients with both cancer and non-cancer pain.[27, 28] Ratings of pain interference with various activities increased as ratings of pain severity were higher. The proportion of patients receiving opioid analgesics also increased with increased severity rating.

The BPI uses 0 to 10 numeric rating scales for item rating because of its simplicity and lack of ambiguity. Since pain can be variable over a day, the BPI asks patients to rate their pain at the time of completing the questionnaire, and also at its worst, least, and average over the previous 7 days. The primary endpoint for this clinical trial will be based on the 7-day worst joint pain as reported on the BPI. The ratings can be combined to give a composite index of pain severity. Also, using numeric 0 to 10 scales, with 0 being “no interference” and 10 being “interferes completely”, the BPI asks for ratings of the degree to which pain interferes with mood, walking and other physical activity, works, social activity, relations with others, and sleep. The mean of these scores can be used as a pain interference score. This will be completed at baseline, 6, and 12 weeks (and off study if patient discontinues before week 12).

PROMIS-29+2 Profile v2.1: A validated questionnaire that assesses patient-reported symptoms over the past 7 days in 9 PROMIS domains (fatigue, sleep disturbance, physical functioning, depression, anxiety, ability to participate in social roles and activities, cognitive function-abilities, and pain intensity and interference). Raw scores for each domain are calculated and then converted to a

T-score, with a mean of 50 and a standard deviation of 10. Higher T scores represent more of the concept being measured.[24] This 31-item questionnaire will be completed at baseline, 6 and 12 weeks (and off study if patient discontinues before week 12).

Global Ratings of Change (GRC) Scale: This is a single-item measure to assess the overall change in pain and stiffness since starting study treatment. It is a 7-point Likert scale from -3 to +3. This will be completed at 6 and 12 weeks (and off study if patient discontinues before week 12).

Mao Expectancy of Treatment Effect (METE): The METE is a validated 4-item self-report questionnaire rated on a scale of 1-5 (from total disagreement to total agreement), which assesses a patient's expectation that acupressure will relieve her AIMSS symptoms.[25] The instrument had good internal consistency (Cronbach's α 0.95) in a prior study of acupuncture in patients with cancer.[29] Will be completed at baseline.

Fibromyalgia (FM) Survey: The FM survey is a combined measure of widespread pain (body map of painful sites) and symptom severity (e.g., fatigue, cognitive problems, headache, poor mood, scores range 0-12), as a self-reported proxy of centralized pain, assessed at baseline. This is a continuous measure with scores ranging from 0-31.[30, 31] Will be completed at baseline.

Massachusetts General Hospital Acupuncture Sensation Scale (MASS): consists of twelve predefined descriptors and one subjective component specified by the subjects in their own words to measure levels of *de qi* sensations. *De qi* is a prerequisite term in Traditional Chinese Medicine used to describe sensory stimulation. It has historically been thought to be an indicator of appropriate acupuncture or acupressure treatment and thus relates to therapeutic effectiveness.[32]

In addition to these validated questionnaires, two non-validated surveys will be used to collect information:

Demographics Questionnaire: This 5-item survey will be used to collect basic information about sex, race/ethnicity, education, marital status, and income. Participants are given the option of not answering each of the questions.

Post-intervention Survey: This non-validated survey will ask participants what type of acupressure they thought they were performing (relaxing acupressure or sham acupressure), whether they felt the benefits they received were worth the time spent performing acupressure, whether they would recommend this intervention to others, and whether they used the AcuWand device.

9.4 Stool samples

9.4.1 Collection

Patients will be given the necessary supplies to collect stool samples using manufacturer protocols and mail them to the cancer center using UPS or USPS mailers (see Appendix B and C).

9.4.2 Method

Samples will be analyzed according to standard methods by the UM Microbiome Core. The method to be used is their standard 16S protocol that targets and amplifies the V4 region of bacterial and archaeal 16S rRNA-encoding genes.[33]

10 CRITERIA FOR EVALUATION AND ENDPOINT

10.1 Safety

Few adverse events are expected in this non-pharmacologic, non-invasive interventional study. Routine safety and tolerability will be evaluated by the study coordinator.

10.2 Stopping Rules

N/A

11 STATISTICAL CONSIDERATIONS

11.1 Statistical hypothesis

Use of true self-acupressure for 12 weeks in postmenopausal women with breast cancer who are experiencing AIMSS will result in improvement in joint pain compared to sham self-acupressure for 12 weeks.

11.2 Sample size determination

With a sample size of 44 evaluable participants (22 evaluable per arm), assuming a standard deviation of difference in worst pain score between treatment groups of 2.3 points, we will have 80% power to detect a reduction of 2 points in worst pain on the BPI at 12 weeks,[1] assuming a 5% significance level. A 2-point improvement in pain is considered a minimal clinically important difference.[1] A total of 50 participants will be enrolled to account for potential dropout, to ensure 44 participants are evaluable for the primary endpoint.

11.3 Statistical Analyses

11.3.1 Primary endpoint

All demographic, clinical and outcome variables will be described using means, standard deviations, frequencies and proportions for the full sample and by treatment group.

The primary endpoint is change in worst joint pain (range 0-10) with 12 weeks of the intervention. A linear model using generalized estimating equations with robust variance will be used with a treatment group, time and a time by group interaction to determine the difference between true acupressure and sham acupressure. We will use an identity link function employing a transformation if needed on the outcome. We will assess the success of blinding by comparing the proportion of participants

who believed they had received true acupressure across groups using a chi-square test.

12.3.2 Secondary endpoints

1. The proportion of patients who have at least a 2-point reduction in *worst and average* pain from baseline to 12 weeks will be reported with the corresponding exact binomial confidence intervals in each treatment group. The proportions will be compared using a chi-square test for each outcome. Differences from baseline at each time point for worst pain will be summarized via mean and standard deviation. Linear models using generalized estimating equations will assess the change in average and worst pain over time between groups similar to the primary endpoint.

2. Differences from baseline at each time point for pain interference (range 0-10) will be summarized via mean and standard deviation. Linear models using generalized estimating equations will assess the change in pain interference over time between groups similar to the primary endpoint.

3. Descriptive statistics including mean, standard deviation, median, interquartile range, t tests, and chi squared tests will be used to describe all other patient-reported outcomes (sleep disturbance [range 32-73.3], fatigue [range 33.7-75.8], physical function [range 22.5-57], anxiety [range 40.3-81.6], depression [range 41-79.4], and cognitive function [range 29.4-61.2] over time), as appropriate. Patient reported outcomes will be analyzed using linear models and generalized estimating equations with robust variance similar to the primary outcome. Normalizing transformations will be used if necessary to ensure accurate model fit.
4. Adherence to the intervention will be assessed through the trial and reported using descriptive statistics. Adherence will be calculated based on number of days acupressure is performed and number of minutes of acupressure performed each day. The proportion of participants who are fully adherent will be compared between groups using a chi-square test.

5. Safety will be assessed throughout the trial and adverse events will be reported using descriptive statistics.

11.3.2 Exploratory endpoints

1. Association between expectation of response to acupressure based on the METE questionnaire and observed response based on change in BPI worst pain score will be analyzed using Spearman correlation.

2. The gut microbiota at baseline and after 12 weeks will be analyzed by group. Change in bacterial phyla and genera proportions and Shannon diversity indices over time will be examined using Wilcoxon rank sum tests to compare groups. Principal components analysis will be conducted to display the microbiome space between compare groups. Differences in structure among groups will be assessed using PERMANOVA (permutational multivariate analysis of variance).

We plan to reduce missing data through monitoring of submission of electronic patient-reported outcomes data and reaching out to participants via text, email, or phone as noted above. We will record all reasons for study withdrawal and missing data. We will assess missing data across study groups, covariates and time points and use multiple imputation via chained equations if it is appreciable.

12 DATA SUBMISSION SCHEDULE

The Case Report Forms (CRFs) are a set of (electronic or paper) forms for each patient that provides a record of the data generated according to the protocol. These forms will be completed on an on-going basis during the study. The medical records and self-report from the patient, when appropriate, will be source of verification of the data. The CRFs will be completed by the Investigator or a member of the study team as listed on the Delegation of Duties Log.

13 SPECIAL INSTRUCTIONS

13.1 Patient-reported questionnaires

Prior to the first time the patient completes the questionnaires: Patients should be directed to report all symptoms and limitations whether or not they are related to the cancer or its treatment. Discourage family members from influencing patient responses to the questions. They should also be told that their treating provider will not be told about symptoms that they report on the questionnaires, and if they are bothersome they should mention them to their treatment team.

Whenever possible, questionnaires will be completed online, although if a participant states that they are unable or unwilling to complete the questionnaire online then the questionnaires can be completed by phone.

Participants will be sent notifications, texts, or emails, as appropriate, that questionnaires are available to questionnaires 2 days before each scheduled assessment time at baseline and weeks 6 and 12. If they have not completed the questionnaire by the scheduled assessment time they will be sent a reminder by text, email and/or phone. If they do not answer the questions by 2 days after the assessment they will be contacted by the study coordinator to complete them by phone.

14 ETHICAL AND REGULATORY CONSIDERATIONS

14.1 Informed consent

Informed consent will be obtained from all research participants prior to performing any study procedures using the most recent IRB-approved version.

14.2 Human Subjects Protections

14.2.1 Rationale for Subject Selection

This clinical trial is open to all patients with AIMSS with minimal restrictions in order to permit inclusion of as broad a population as possible. Only patients who read English will be enrolled because the study app is not available in other languages.

14.2.2 Participation of Children

Patients must be at least 18 years of age to participate.

14.3 Institutional Review

Before implementing this study, the protocol, the proposed informed consent form and other information to be provided to subjects, must be reviewed and approved by a properly constituted IRB. Any amendments to the protocol must be reviewed and approved by the IRB.

14.4 Data and Safety Monitoring Plan

This study will be monitored in accordance with the NCI approved University of Michigan Rogel Cancer Center Data and Safety Monitoring Plan.

The study team will meet every six months or more frequently depending on the activity of the protocol. The discussion will include matters related to the safety of study participants (SAE/UaP reporting), validity and integrity of the data, enrollment rate relative to expectations, characteristics of participants, retention of participants, adherence to the protocol (potential or real protocol deviations) and data completeness. At these regular meetings, the protocol specific Data and Safety Monitoring Report form will be completed and signed by the Principal Investigator or by one of the co-investigators.

Data and Safety Monitoring Reports will be submitted to the University of Michigan Rogel Cancer Center Data and Safety Monitoring Committee (DSMC) every six months for independent review.

14.5 Adverse Events

14.5.1 Experimental Therapy

Few adverse events are expected from this non-pharmacologic, non-invasive study intervention.

14.5.2 Adverse Event Reporting Requirements

Adverse event (AE) monitoring and reporting is a routine part of every clinical trial and is done to ensure the safety of subjects enrolled in the studies as well as those who will enroll in future studies using similar agents. Data on adverse events will be collected from the time of the initial study treatment through the last administration of study treatment. Any serious adverse event that occurs after

the last study treatment and is considered related to the study treatment must also be reported. Serious Adverse Events (SAEs) will continue to be followed until:

- Resolution or the symptoms or signs that constitute the serious adverse event return to baseline;
- There is satisfactory explanation other than the study treatment for the changes observed; or
- Death.

The investigator is responsible for the detection, documentation, grading and assignment of attribution of events meeting the criteria and definition of an AE or SAE. The definitions of AEs and SAEs are given below. It is the responsibility of the principal investigator to ensure that all staff involved in the trial is familiar with the content of this section.

Any medical condition or laboratory abnormality with an onset date before initial study intervention initiation is considered to be pre-existing in nature. Any known pre-existing conditions that are ongoing at time of study entry should be considered medical history.

All events meeting the criteria and definition of an AE or SAE, as defined in Section 15.5.3, occurring from the initial study intervention initiation to the last administration of the study intervention must be recorded as an adverse event in the patient's source documents and on the CRF regardless of frequency, severity (grade) or assessed relationship to the study treatment, except as outlined below.

In addition to new events, any increase in the frequency or severity (i.e., toxicity grade) of a pre-existing condition that occurs after the patient begins study treatment is also considered an adverse event.

14.5.3 Definitions

1. Adverse Event

An adverse event (AE) is any untoward medical occurrence in a patient receiving study treatment and which does not necessarily have a causal relationship with this treatment. An AE can be any unfavorable and unintended sign (including an abnormal laboratory finding), symptom, or disease temporally associated with the use of an experimental intervention, whether or not related to the intervention.

- Diagnostic and therapeutic non-invasive and invasive (i.e., surgical) procedures will not be reported as adverse events. However, the medical condition for which the procedure was performed must be reported if it meets the definition of an adverse event unless it is a pre-existing (prior to protocol treatment) condition.

- Symptoms of the original or targeted disease are not to be considered adverse events for this study. The following symptoms are indicative of underlying disease (breast cancer) or its treatment and will not be reported as adverse events (unless the event is considered serious):
    - AI-associated arthralgias (will be collected on patient-reported questionnaires)
    - Symptoms and signs due to prior treatments (chemotherapy, radiation therapy, surgery)
    - Adverse events related to disease progression
    - Hospitalization or treatment related to breast surgery or breast reconstruction procedures

2. Serious Adverse Event

An adverse event is considered "serious" if, in the view of the investigator, it results in any of the following outcomes:

- Death
  If death results from (progression of) the disease, the disease should be reported as event (SAE) itself.

- A life-threatening adverse event
  An adverse even is considered 'life-threatening' if, in the view of either the investigator, its occurrence places the patient or subject at immediate risk of death. It does not include an adverse event that, had it occurred in a more severe form, might have caused death.

- Inpatient hospitalization or prolongation of existing hospitalization for $\geq$ 24 hours.

- A persistent or significant incapacity or substantial disruption of the ability to conduct normal life functions

- A congenital anomaly/birth defect

- Important medical event
  Any event that may not result in death, be life-threatening, or require hospitalization may be considered serious when, based upon appropriate medical judgment, they may jeopardize the patient and may require medical or surgical intervention to prevent one of the outcomes listed in this definition of "Serious Adverse Event". Examples of such medical events include allergic bronchospasm requiring intensive treatment in an emergency room or at home; convulsions that do not result in inpatient hospitalization or the development of drug dependency or drug abuse.

Previously planned (prior to signing the informed consent form) surgeries should not be reported as SAEs unless the underlying medical condition has worsened during the course of the study. Preplanned hospitalizations or procedures for preexisting conditions that are already recorded in the patient's medical history at the time of study enrollment should not be considered SAEs. Hospitalization or prolongation of hospitalization without a precipitating clinical AE (for example, for the administration of study therapy or other protocol-required procedure) should not be considered SAEs. However, if the preexisting condition worsened during the course of the study, it should be reported as an SAE.

3. Expected Adverse Events

An adverse event (AE) is considered "expected" if it is listed as expected in the protocol and in the consent document.

4. Unexpected Adverse Event

An adverse event (AE) is considered "unexpected" if it is not described in the protocol or in the informed consent document.

14.5.4 Adverse Event Characteristics

a. CTCAE Term

AE description and grade: The descriptions and grading scales found in the NCI Common Terminology Criteria for Adverse Events (CTCAE) version 5.0 will be utilized for AE reporting. All appropriate treatment areas should have access to a copy of the CTCAE version 5.0. A copy of the CTCAE version 5.0 can be downloaded from the CTEP web site. (http://ctep.cancer.gov)

b. Attribution of the AE

The investigator or co-investigator is responsible for assignment of attribution.

Definite – The AE *is clearly related* to the study treatment.
Probable – The AE *is likely related* to the study treatment.
Possible – The AE *may be related* to the study treatment.
Unlikely – The AE *is doubtfully related* to the study treatment.
Unrelated – The AE *is clearly NOT related* to the study treatment.

14.5.5 Serious Adverse Event Reporting Guidelines

All Serious Adverse Events that are deemed related, probably related or possibly related will be reported to the IRBMED per institutional guidelines.

14.5.6 Routine Reporting

All other adverse events will be reported per current institutional guidelines.

14.5.7 Reporting of Unanticipated Problems

There are types of incidents, experiences and outcomes that occur during the conduct of human subjects research that represent unanticipated problems but are not considered adverse events. For example, some unanticipated problems involve social or economic harm instead of the physical or psychological harm associated with adverse events. In other cases, unanticipated problems place subjects or others at increased risk of harm, but no harm occurs.

Upon becoming aware of any incident, experience, or outcome (not related to an adverse event) that may represent an unanticipated problem, the investigator should assess whether the incident, experience, or outcome represents an unanticipated problem. The incident, experience or outcomes is considered unanticipated if it meets all of the following criteria:

1. Unexpected (in terms of nature, severity, or frequency);
2. Related or possibly related to participation in the research; and
3. Suggests that the research places subjects or others at a greater risk of harm than was previously known or recognized.

If the investigator determines that the incident, experience, or outcome represents an unanticipated problem, the investigator must report it to the IRB per institutional guidelines.

14.6 Protocol Amendments

Any amendments or administrative changes in the research protocol during the period, for which the IRB approval has already been given, will not be initiated without submission of an amendment for IRB review and approval.

These requirements for approval will in no way prevent any immediate action from being taken by the investigator in the interests of preserving the safety of all subjects included in the trial.

14.7 Protocol Deviations

A protocol deviation (or violation) is any departure from the defined procedures and treatment plans as outlined in the approved protocol version. Protocol deviations have the potential to place participants at risk and can also undermine the scientific integrity of the study thus jeopardizing the justification for the research. All deviations will be reported to the IRB per current institutional guidelines.

14.8 Clinical Trials Data Bank

The study will be registered on **http://clinicaltrials.gov**

15 REFERENCES

1. Dworkin RH, Turk DC, Wyrwich KW, et al. Interpreting the clinical importance of treatment outcomes in chronic pain clinical trials: IMMPACT recommendations. *J Pain*. Feb 2008;9(2):105-21. doi:S1526-5900(07)00899-1 [pii]
10.1016/j.jpain.2007.09.005
2. Burstein HJ, Lacchetti C, Anderson H, et al. Adjuvant Endocrine Therapy for Women With Hormone Receptor-Positive Breast Cancer: ASCO Clinical Practice Guideline Focused Update. *J Clin Oncol*. Feb 10 2019;37(5):423-438. doi:10.1200/JCO.18.01160
3. Smith IE, Dowsett M. Aromatase inhibitors in breast cancer. *N Engl J Med*. Jun 12 2003;348(24):2431-42.
4. Dowsett M, Cuzick J, Ingle J, et al. Meta-analysis of breast cancer outcomes in adjuvant trials of aromatase inhibitors versus tamoxifen. *J Clin Oncol*. Jan 20 2010;28(3):509-18. doi:10.1200/JCO.2009.23.1274
5. Henry NL, Azzouz F, Desta Z, et al. Predictors of aromatase inhibitor discontinuation due to treatment-emergent symptoms in early-stage breast cancer. *J Clin Oncol*. 2012;30(9):936-942.
6. Mao JJ, Stricker C, Bruner D, et al. Patterns and risk factors associated with aromatase inhibitor-related arthralgia among breast cancer survivors. *Cancer*. Aug 15 2009;115(16):3631-9. doi:10.1002/cncr.24419
7. Henry NL, Conlon A, Kidwell KM, et al. Effect of estrogen depletion on pain sensitivity in aromatase inhibitor-treated women with early-stage breast cancer. *J Pain*. May 2014;15(5):468-75. doi:10.1016/j.jpain.2014.01.487
8. Henry NL, Pchejetski D, A'Hern R, et al. Inflammatory cytokines and aromatase inhibitor-associated musculoskeletal syndrome: a case-control study. *Br J Cancer*. Jul 27 2010;103(3):291-6. doi:10.1038/sj.bjc.6605768
9. Henry NL, Skaar TC, Dantzer J, et al. Genetic associations with toxicity-related discontinuation of aromatase inhibitor therapy for breast cancer. *Breast Cancer Res Treat*. Apr 2013;138(3):807-16. doi:10.1007/s10549-013-2504-3
10. Mao JJ, Su HI, Feng R, et al. Association of functional polymorphisms in CYP19A1 with aromatase inhibitor associated arthralgia in breast cancer survivors. *Breast Cancer Res*. Jan 20 2011;13(1):R8. doi:10.1186/bcr2813
11. Niravath P. Aromatase inhibitor-induced arthralgia: a review. *Ann Oncol*. Jun 2013;24(6):1443-9. doi:10.1093/annonc/mdt037
12. Bauml J, Chen L, Chen J, et al. Arthralgia among women taking aromatase inhibitors: is there a shared inflammatory mechanism with co-morbid fatigue and insomnia? *Breast Cancer Res*. Jun 28 2015;17:89. doi:10.1186/s13058-015-0599-7
13. Henry NL, Unger JM, Schott AF, et al. Randomized, multicenter, placebo-controlled clinical trial of duloxetine versus placebo for aromatase inhibitor-associated arthralgias in early-stage breast cancer: SWOG S1202. *J Clin Oncol*. Feb 1 2018;36(4):326-332. doi:10.1200/JCO.2017.74.6651
14. Hershman DL, Unger JM, Greenlee H, et al. Effect of acupuncture vs sham acupuncture or waitlist control on joint pain related to aromatase inhibitors among women with early-stage breast cancer: a randomized clinical trial. *JAMA*. Jul 10 2018;320(2):167-176. doi:10.1001/jama.2018.8907

15. Chirgwin JH, Giobbie-Hurder A, Coates AS, et al. Treatment adherence and its impact on disease-free survival in the Breast International Group 1-98 trial of tamoxifen and letrozole, alone and in sequence. *J Clin Oncol*. Jul 20 2016;34(21):2452-9. doi:10.1200/JCO.2015.63.8619
16. Hershman DL, Shao T, Kushi LH, et al. Early discontinuation and non-adherence to adjuvant hormonal therapy are associated with increased mortality in women with breast cancer. *Breast Cancer Res Treat*. Apr 2011;126(2):529-37. doi:10.1007/s10549-010-1132-4
17. Barron TI, Cahir C, Sharp L, Bennett K. A nested case-control study of adjuvant hormonal therapy persistence and compliance, and early breast cancer recurrence in women with stage I-III breast cancer. *Br J Cancer*. Sep 17 2013;109(6):1513-21. doi:10.1038/bjc.2013.518
18. Greenlee H, Crew KD, Capodice J, et al. Methods to Standardize a Multicenter Acupuncture Trial Protocol to Reduce Aromatase Inhibitor-related Joint Symptoms in Breast Cancer Patients. *J Acupunct Meridian Stud*. Jun 2015;8(3):152-8. doi:10.1016/j.jams.2015.03.006
19. Zick SM, Sen A, Wyatt GK, Murphy SL, Arnedt JT, Harris RE. Investigation of 2 Types of Self-administered Acupressure for Persistent Cancer-Related Fatigue in Breast Cancer Survivors: A Randomized Clinical Trial. *JAMA Oncol*. Nov 1 2016;2(11):1470-1476. doi:10.1001/jamaoncol.2016.1867
20. Zick SM, Harris RE. The Effect of Self-Administered Acupressure on Chronic Pain in Breast Cancer Survivors. *The Journal of Alternative and Complementary Medicine*. May 2014;20(5):A23. doi:doi:10.1089/acm.2014.5057.abstract.
21. Zick SM. Relaxation Acupressure Reduces Persistent Cancer-Related Fatigue. *Evidence-based complementary and alternative medicine*. 2011;165(2):1-11. doi:10.1155/2011/142913
22. Lichstein KL, Means MK, Noe SL, Aguillard RN. Fatigue and sleep disorders. *Behaviour research and therapy*. Aug 1997;35(8):733-40.
23. Daut RL, Cleeland CS, Flanery RC. Development of the Wisconsin Brief Pain Questionnaire to assess pain in cancer and other diseases. *Pain*. Oct 1983;17(2):197-210.
24. Cella D, Riley W, Stone A, et al. The Patient-Reported Outcomes Measurement Information System (PROMIS) developed and tested its first wave of adult self-reported health outcome item banks: 2005-2008. *J Clin Epidemiol*. Nov 2010;63(11):1179-94. doi:S0895-4356(10)00173-3 [pii]
10.1016/j.jclinepi.2010.04.011
25. Keefe JR, Amsterdam J, Li QS, Soeller I, DeRubeis R, Mao JJ. Specific expectancies are associated with symptomatic outcomes and side effect burden in a trial of chamomile extract for generalized anxiety disorder. *J Psychiatr Res*. Jan 2017;84:90-97. doi:10.1016/j.jpsychires.2016.09.029
26. Wyatt GK, Frambes DA, Harris RE, Arnedt JT, Murphy SL, Zick SM. Self-administered Acupressure for Persistent Cancer-related Fatigue: Fidelity Considerations. *Altern Ther Health Med*. Jul-Aug 2015;21(4):18-23.
27. Tan G, Jensen MP, Thornby JI, Shanti BF. Validation of the Brief Pain Inventory for chronic nonmalignant pain. *J Pain*. Mar 2004;5(2):133-7.
28. Tittle MB, McMillan SC, Hagan S. Validating the brief pain inventory for use with surgical patients with cancer. *Oncology nursing forum*. Mar-Apr 2003;30(2):325-30.
29. Mao JJ, Xie SX, Bowman MA. Uncovering the expectancy effect: the validation of the acupuncture expectancy scale. *Altern Ther Health Med*. Nov-Dec 2010;16(6):22-7.

30. Wolfe F, Clauw DJ, Fitzcharles MA, et al. Fibromyalgia criteria and severity scales for clinical and epidemiological studies: a modification of the ACR Preliminary Diagnostic Criteria for Fibromyalgia. *J Rheumatol*. Jun 2011;38(6):1113-22. doi:jrheum.100594 [pii] 10.3899/jrheum.100594
31. Wolfe F. Fibromyalgianess. *Arthritis Rheum*. Jun 15 2009;61(6):715-6. doi:10.1002/art.24553
32. Jang JC, Jung J, Lee H, Park YB, Kim H. Multidimensional Aspects of de qi Sensations in MASS and ASQ Assessment: A Pilot Study. *Evid Based Complement Alternat Med*. 2017;2017:6249329. doi:10.1155/2017/6249329
33. Kozich JJ, Westcott SL, Baxter NT, Highlander SK, Schloss PD. Development of a dual-index sequencing strategy and curation pipeline for analyzing amplicon sequence data on the MiSeq Illumina sequencing platform. *Appl Environ Microbiol*. Sep 2013;79(17):5112-20. doi:10.1128/AEM.01043-13

16 Appendices

16.1 Appendix A: Patient-Reported Outcomes Questionnaires

1. Brief Pain Inventory
2. Demographics questionnaire
3. Fibromyalgia Survey
4. Global Ratings Scale (GRC) for joint pain and joint stiffness
5. Massachusetts General Hospital Acupuncture Sensation Scale (MASS)
6. METE-acupressure
7. Post-intervention survey
8. PROMIS 29+2 Profile

16.2 Appendix B: Instructions for Collecting a Stool Sample at Home – UPS

16. 3 Appendix C: Instructions for Collecting a Stool Sample at Home - USPS

Brief Pain Inventory (BPI)

For each item below, please circle the number that best describes your pain and how that pain affects your daily activities. Be sure to read each question carefully and to provide one answer for each question. Please think about joint pain when answering these questions. If you had no joint pain then you should answer “no” or “0”. Thank you for participating in this study.

1. Throughout our lives, most of us have had pain from time to time (such as minor headaches, sprains, and toothaches). Have you had pain other than these everyday kinds of pain in the last week? ☐ Yes ☐ No

2. Please rate your pain by circling the one number that best describes your pain at its **WORST** in the last week.

0	1	2	3	4	5	6	7	8	9	10
No joint pain										Pain as bad as you can imagine

3. Please rate your pain by circling the one number that best describes your pain at its **LEAST** in the last week.

0	1	2	3	4	5	6	7	8	9	10
No joint pain										Pain as bad as you can imagine

4. Please rate your pain by circling the one number that best describes your pain on the **AVERAGE** in the last week.

0	1	2	3	4	5	6	7	8	9	10
No joint pain										Pain as bad as you can imagine

5. Please rate your pain by circling the one number that tells how much pain you have **RIGHT NOW**.

0	1	2	3	4	5	6	7	8	9	10
No joint pain										Pain as bad as you can imagine

6. Are you taking any oral medications for your pain?

☐ Yes ☐ No

7. In the past week, how much **RELIEF** have pain treatments or medications provided? Please circle the one percentage that most shows how much.

0%	10%	20%	30%	40%	50%	60%	70%	80%	90%	100%
No relief										Complete relief

8. Circle the one number that describes how, during the last week, **PAIN HAS INTERFERED** with your:

A. **General activity**

0	1	2	3	4	5	6	7	8	9	10
Does not interfere										Completely interferes

B. **Mood**

0	1	2	3	4	5	6	7	8	9	10
Does not interfere										Completely interferes

C. **Walking ability**

0	1	2	3	4	5	6	7	8	9	10
Does not interfere										Completely interferes

D. **Normal work** (includes both work outside the home and housework)

0	1	2	3	4	5	6	7	8	9	10
Does not interfere										Completely interferes

E. **Relations with other people**

0	1	2	3	4	5	6	7	8	9	10
Does not interfere										Completely interferes

F. **Sleep**

0	1	2	3	4	5	6	7	8	9	10
Does not interfere										Completely interferes

G. **Enjoyment of life**

0	1	2	3	4	5	6	7	8	9	10
Does not interfere										Completely interferes

Source: Brief Pain Inventory-Short Form: C. S. Cleeland, PhD, University of Texas, M. D. Anderson Cancer Center, Houston, TC (Cleeland, 1994)

Acu-AIM Demographics Questionnaire

The following questions are some general questions about you.

1. What is your sex?
___ (1) Female
___ (2) Male
___ (3) Intersex
___ (4) Prefer not to answer

2. How do you describe your ethnicity/race (check all that apply)?
___ (1) White
___ (2) Black or African American
___ (3) Asian
___ (4) Hispanic or Latino
___ (5) American Indian/Alaska Native
___ (6) Native Hawaiian or other Pacific Islander
___ (7) Prefer not to answer

3. What is your marital status?
___ (1) Single
___ (2) Married
___ (3) Divorced
___ (4) Separated
___ (5) Widowed
___ (6) Committed relationship
___ (7) Prefer not to answer

4. How much education have you completed?
___ (1) High school or less
___ (2) Some college
___ (3) College graduate
___ (4) Postgraduate education
___ (5) Prefer not to answer

5. What is your yearly household income?
___ (1) $30,000 or less
___ (2) $30,001 - $60,000
___ (3) $60,001 - $100,000
___ (4) $100,001 or more
___ (5) Prefer not to answer

Fibromyalgia Survey

Check each area you have felt pain in over the past week.

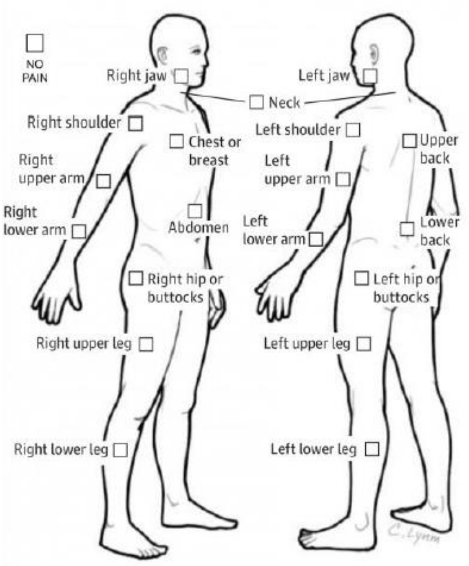

Symptom Severity Scale

Using the following scale, indicate for each item your severity over the **past week** by checking the appropriate box:

	No problem	Slight or mild problem: generally mild or intermittent	Moderate problem: considerable problems; often present and/or at a moderate level	Severe problem: continuous, life-disturbing problems
Fatigue	○	○	○	○
Trouble thinking or remembering	○	○	○	○
Waking up tired (unrefreshed)	○	○	○	○

During the **past 6 months**, have you had any of the following symptoms?

	Yes	No
Pain or cramps in lower abdomen	○	○
Depression	○	○
Headache	○	○

GLOBAL RATING OF CHANGE

Joint pain

Thinking about pain in your joints, please use the scale below to indicate whether there has been any change since you started using acupressure.

Since you started using acupressure, your level of joint pain is... (check one box below)

- ☐ Very much better
- ☐ Moderately better
- ☐ A little better
- ☐ About the same
- ☐ A little worse
- ☐ Moderately worse
- ☐ Very much worse

Joint stiffness

Thinking about stiffness in your joints, please use the scale below to indicate whether there has been any change since you started using acupressure.

Since you started using acupressure, your level of joint stiffness is... (check one box below)

- ☐ Very much better
- ☐ Moderately better
- ☐ A little better
- ☐ About the same
- ☐ A little worse
- ☐ Moderately worse
- ☐ Very much worse

MASS

Take a moment to recall any de qi sensations you experienced during acupressure. On the next form, there is a list of words often used to describe de qi sensations. Because the experience of de qi is different for different people, these words may or may not describe what you felt. If you felt something that is not listed, please write it on the blank marked “OTHER.” If you do not experience any sensations, you can mark “0” (no sensation) for all items, including “OTHER.” Now, please mark to what extent these words describe what you experienced during acupressure.

[Kong et al, *J Alternative Complementary* Med 2007; Adapted for acupressure]

	0	1	2	3	4	5	6	7	8	9	10
soreness	none		mild			moderate			strong		unbearable
aching	none		mild			moderate			strong		unbearable
deep pressure	none		mild			moderate			strong		unbearable
heaviness	none		mild			moderate			strong		unbearable
fullness/distention	none		mild			moderate			strong		unbearable
tingling	none		mild			moderate			strong		unbearable
numbness	none		mild			moderate			strong		unbearable
sharp pain	none		mild			moderate			strong		unbearable
dull pain	none		mild			moderate			strong		unbearable
warmth	none		mild			moderate			strong		unbearable
cold	none		mild			moderate			strong		unbearable
throbbing	none		mild			moderate			strong		unbearable
other (subject defined)	none		mild			moderate			strong		unbearable

METE

Each individual may have a different expectation for the effects of acupressure. If we use the following sentences to describe **your expectation** of **acupressure's** effect on your **joint pain and stiffness** after the entire course of acupressure therapy, how much do you **agree**?

For each statement, please pick the closest answer by checking the box:

	Not at all Agree	A Little Agree	Moderately Agree	Mostly Agree	Completely Agree
1. My joint pain and stiffness will improve a lot.	☐	☐	☐	☐	☐
2. I will be able to cope with my joint pain and stiffness better.	☐	☐	☐	☐	☐
3. The symptoms of my joint pain and stiffness will disappear.	☐	☐	☐	☐	☐
4. My energy level will increase.	☐	☐	☐	☐	☐

Post-Intervention Survey

1. Do you think you were assigned to the true acupressure or sham acupressure group?
   - ☐ True acupressure
   - ☐ Sham acupressure
   - ☐ Not sure

2. Which of the following statements do you agree with most?

☐ The study treatment was beneficial and it took a reasonable amount of time to do the acupressure each day

☐ The study treatment was beneficial despite how long it took to do the acupressure each day

☐ The symptom improvement wasn't worth the time it took to do the acupressure each day

☐ I didn't experience any improvement in symptoms

☐ None of the above

☐ Prefer not to answer

3. Did you use the acupressure points described in the study app (on the tablet), or did you use other acupressure points that you found online or in another source?
   - ☐ Acupoints in the app
   - ☐ Acupoints from another source
   - ☐ Not sure

4. Would you recommend acupressure to others who are having similar symptoms?
   - ☐ Yes
   - ☐ No
   - ☐ Not sure

5. What device did you use to perform the acupressure treatment?
   - ☐ AcuWand
   - ☐ Pencil eraser (or similar)
   - ☐ Fingertip
   - ☐ Other

6. If you used the AcuWand, do you think it was helpful for administering acupressure?
   - ☐ Yes
   - ☐ No
   - ☐ Not sure
   - ☐ Did not use AcuWand

PROMIS 29+2 Profile v2.1

Please respond to each question or statement by marking one box per row.

	Physical Function	Without any difficulty	With a little difficulty	With some difficulty	With much difficulty	Unable to do
PFA11	Are you able to do chores such as vacuuming or yard work?	☐ 5	☐ 4	☐ 3	☐ 2	☐ 1
PFA21	Are you able to go up and down stairs at a normal pace?	☐ 5	☐ 4	☐ 3	☐ 2	☐ 1
PFA23	Are you able to go for a walk of at least 15 minutes?	☐ 5	☐ 4	☐ 3	☐ 2	☐ 1
PFA53	Are you able to run errands and shop?	☐ 5	☐ 4	☐ 3	☐ 2	☐ 1

	Anxiety<br>In the past 7 days…	Never	Rarely	Sometimes	Often	Always
EDANX01	I felt fearful	☐ 1	☐ 2	☐ 3	☐ 4	☐ 5
EDANX40	I found it hard to focus on anything other than my anxiety	☐ 1	☐ 2	☐ 3	☐ 4	☐ 5
EDANX41	My worries overwhelmed me	☐ 1	☐ 2	☐ 3	☐ 4	☐ 5
EDANX53	I felt uneasy	☐ 1	☐ 2	☐ 3	☐ 4	☐ 5

	Depression<br>In the past 7 days...	Never	Rarely	Sometimes	Often	Always
EDDEP04	I felt worthless	☐ 1	☐ 2	☐ 3	☐ 4	☐ 5
EDDEP06	I felt helpless	☐ 1	☐ 2	☐ 3	☐ 4	☐ 5
EDDEP29	I felt depressed	☐ 1	☐ 2	☐ 3	☐ 4	☐ 5
EDDEP41	I felt hopeless	☐ 1	☐ 2	☐ 3	☐ 4	☐ 5

	Fatigue<br>During the past 7 days…	Not at all	A little bit	Somewhat	Quite a bit	Very much
HI7	I feel fatigued	☐ 1	☐ 2	☐ 3	☐ 4	☐ 5
AN3	I have trouble starting things because I am tired	☐ 1	☐ 2	☐ 3	☐ 4	☐ 5

	**Fatigue**<br>**In the past 7 days…**	**Not at all**	**A little bit**	**Somewhat**	**Quite a bit**	**Very much**
FATEXP41	How run-down did you feel on average?	☐ 1	☐ 2	☐ 3	☐ 4	☐ 5
FATEXP40	How fatigued were you on average?	☐ 1	☐ 2	☐ 3	☐ 4	☐ 5
	**Sleep Disturbance**<br>**In the past 7 days…**	**Very poor**	**Poor**	**Fair**	**Good**	**Very good**
Sleep109	My sleep quality was	☐ 5	☐ 4	☐ 3	☐ 2	☐ 1
	**In the past 7 days…**	**Not at all**	**A little bit**	**Somewhat**	**Quite a bit**	**Very much**
Sleep116	My sleep was refreshing.	☐ 5	☐ 4	☐ 3	☐ 2	☐ 1
Sleep20	I had a problem with my sleep	☐ 1	☐ 2	☐ 3	☐ 4	☐ 5
Sleep44	I had difficulty falling asleep	☐ 1	☐ 2	☐ 3	☐ 4	☐ 5
	**Ability to Participate in Social Roles and Activities**	**Never**	**Rarely**	**Sometimes**	**Usually**	**Always**
SRPPER11_CaPS	I have trouble doing all of my regular leisure activities with others	☐ 5	☐ 4	☐ 3	☐ 2	☐ 1
SRPPER18_CaPS	I have trouble doing all of the family activities that I want to do	☐ 5	☐ 4	☐ 3	☐ 2	☐ 1
SRPPER23_CaPS	I have trouble doing all of my usual work (include work at home)	☐ 5	☐ 4	☐ 3	☐ 2	☐ 1
SRPPER46_CaPS	I have trouble doing all of the activities with friends that I want to do	☐ 5	☐ 4	☐ 3	☐ 2	☐ 1
	**Pain Interference**<br>**In the past 7 days…**	**Not at all**	**A little bit**	**Somewhat**	**Quite a bit**	**Very much**
PAININ9	How much did pain interfere with your day to day activities?	☐ 1	☐ 2	☐ 3	☐ 4	☐ 5
PAININ22	How much did pain interfere with work around the home?	☐ 1	☐ 2	☐ 3	☐ 4	☐ 5
PAININ31	How much did pain interfere with your ability to participate in social activities?	☐ 1	☐ 2	☐ 3	☐ 4	☐ 5

	Pain Interference<br>In the past 7 days…	Not at all	A little bit	Somewhat	Quite a bit	Very much
PAININ34	How much did pain interfere with your household chores?	☐ 1	☐ 2	☐ 3	☐ 4	☐ 5

	Cognitive Function - Abilities<br>In the past 7 days…	Not at all	A little bit	Somewhat	Quite a bit	Very much
PC6r	I have been able to concentrate	☐ 1	☐ 2	☐ 3	☐ 4	☐ 5
PC27r	I have been able to remember to do things, like take medicine or buy something I needed.................	☐ 1	☐ 2	☐ 3	☐ 4	☐ 5

	Pain Intensity<br>In the past 7 days…											
Global07	How would you rate your pain on average?......................................	☐ 0<br>No pain	☐ 1	☐ 2	☐ 3	☐ 4	☐ 5	☐ 6	☐ 7	☐ 8	☐ 9	☐ 10<br>Worst pain imaginable

16.2 Appendix B: Instructions for Collecting a Stool Sample at Home - UPS

Genotek OMNIgene-Gut Instructions

Thank you for participating in our study.
Please read the instructions below for how to collect and return your samples.

Before collecting your stool sample, please verify that all items listed below are included in your kit:

Stool Collection Kit Items:

A. 1 purple and yellow-capped omnigene-gut tube (contains liquid and a mixing ball)

See diagram to the right for clarification.

Do not spill or pour out the liquid that is inside the tube.

B. Spoon/spatula

See diagram to the right

C. Bio-specimen bag
D. Toilet hat for stool collection
E. Cardboard shipping envelope

Picture of purple and yellow-capped omnigene gut tube (1) and spoon (2)

Figure of Collection kit package

<u>Collection tips</u>:

1. Empty your bladder prior to collection.
2. Place the collection hat under the toilet seat.
3. Have a bowel movement. Be sure to collect only stool in the hat (no urine or toilet paper)
4. Toilet paper or tissues may be required.

<u>Collection directions</u>:

1. While holding the yellow tube top**, UNSCREW THE PURPLE TOP ONLY** and set the top aside.

NOTE: <u>do NOT remove the yellow</u> tube top.
NOTE: do NOT spill the liquid that is already in the yellow tube.

2. Transfer one spoonful of solid fecal sample into the yellow tube top. Scrape horizontally across the top of the yellow tube to remove excess. Clean the sides of the container with a paper towel as necessary.

NOTE: Even if the fecal sample doesn't flow through the yellow tube top, it is still OK to proceed to step 3.

3. Pick up the purple cap with the solid end facing **DOWN** and screw onto the yellow tube top until tightly closed.

4. Shake the sealed tube back and forth as hard and fast as possible for a minimum of 30 seconds. Not all particles will dissolve but if there are large particles, continue to shake (see figures A and B for large vs. small particles).

5. Place spoon in original packaging or wrap in toilet paper and discard.

6. Discard the collection hat appropriately. A new one will be sent for any future samples.

7. Write the date you collected your stool on the sticky label located on the biohazard bag.

8. On the second sticky note on the biohazard bag, please mark the one number that best describes your stool from the picture to the right (figure C).

9. Place the sample into the biohazard bag. See "after collection" for steps on mailing the sample.

<u>After collection</u>:

1. Wrap biohazard bag containing your sample in bubble wrap and place into the cardboard mailing envelope.
2. Seal the cardboard mailing envelope (with return label) and bring to a UPS store, UPS drobox, or arrange for a UPS pickup at your home (see "how to schedule a UPS pickup").

<u>How to schedule a UPS pick up</u>:

Call the UPS automated number: 1-800-PICK-UPS (742-5877) to schedule a time for UPS to come and pick up your specimen in the 2-way mailing box.

Any questions or concerns about the process, please contact the study coordinator:

Name: [XXX]

Email: [XXX]

16. 3 Appendix C: Instructions for Collecting a Stool Sample at Home - USPS

Genotek OMNIgene-Gut Instructions

Thank you for participating in our study.
Please read the instructions below for how to collect and return your samples.

Before collecting your stool sample, please verify that all items listed below are included in your kit:

Stool Collection Kit Items:

A. 1 purple and yellow-capped omnigene-gut tube (contains liquid and a mixing ball)

See diagram to the right for clarification.

Do not spill or pour out the liquid that is inside the tube.

B. Spoon/spatula

See diagram to the right

C. Bio-specimen bag

D. Toilet hat for stool collection

E. Cardboard shipping envelope

Picture of Purple and yellow-capped omnigene gut tube (1) and spoon (2)

Picture of Collection kit package

Collection tips:

1. Empty your bladder prior to collection.
2. Place the collection hat under the toilet seat.
3. Have a bowel movement. Be sure to collect only stool in the hat (no urine or toilet paper)
4. Toilet paper or tissues may be required.

Collection directions:

4. Shake the sealed tube back and forth as hard and fast as possible for a minimum of 30 seconds. Not all particles will dissolve but if there are large particles, continue to shake (see figures A and B for large vs. small particles).

5. Place spoon in original packaging or wrap in toilet paper and discard.

6. Discard the collection hat appropriately. A new one will be sent for any future samples.

7. Write the date you collected your stool sample on the sticky label.

8. On the second sticky note on the biohazard bag, please mark the one number that best describes your stool from the picture to the right (figure C).

9. Place the sample into the biohazard bag. See "after collection" for steps on mailing the sample.

After collection:

1. Wrap biohazard bag containing your sample in bubble wrap and place into the cardboard mailing envelope.
2. Place sealed cardboard mailing envelope into outer packaging. Outer packaging will have postage and mailing address already attached.
3. Seal the package and mail via USPS.

Any questions or concerns about the process, please contact the study coordinator:
Name: [XXX]
Email: [XXX]
